# Computational fluid dynamics modeling and simulation of nanoparticle-tumor interaction: Systematic literature review

**DOI:** 10.1016/j.csbj.2025.11.013

**Published:** 2025-11-12

**Authors:** Kamogelo M. Mmereke, Adewale O. Oladipo, Tracy Masebe, Fulufhelo J. Nemavhola, Thanyani Pandelani

**Affiliations:** a Department of Mechanical, Bioresources and Biomedical Engineering, School of Engineering and the Built Environment, College of Science, Engineering and Technology University of South Africa, Private Bag X6, South Africa; b Department of Life and Consumer Sciences, College of Agriculture, Environmental Sciences, University of South Africa, Private Bag X6, South Africa; c Department of Mechanical Engineering, Faculty of Engineering and the Built Environment, Durban University of Technology, PO Box 1334, Durban 4000, South Africa

**Keywords:** Fluid dynamics, Nanoparticle, Tumour, Cancer therapy, Pathologies

## Abstract

Cancer therapy mediated by nanoparticles is gaining recognition for shifting the paradigm of targeted and/or personalized cancer therapy. Despite the great promise, only a few nanoformulations have been clinically approved due to the complexities that limit effective and efficient nanodrug development. Moreover, the preparation of cancer nanodrug has not yet been optimized for clinical approval in patient treatment. Computational fluid dynamics (CFD) is a new technique that simulates and analyzes fluid flows and their interactions with surfaces using computer algorithms and numerical analysis. This simulation and modeling tool provides a distinct advantage in understanding tumor-host mechano-biology and mechanisms that help identify the main factors affecting the transport of tumor-targeting nanoagents. Taking these factors into consideration, the advent of computational fluid dynamics simulation and modeling represents a shift in the optimization of cancer nanoagents’ fluidics. This review briefly introduces the fluid mechanism along with its principles and foundations relating to cancer drug delivery. Key components of tumor microenvironments relating to temperature, flow velocity, fluid pressure, and tumor rheology, as well as physicochemical properties of nanoparticles modulating fluid mechanics, were discussed. It also includes a thorough examination of the advantages and challenges of using nanoformulations such as liposomes, polymers, and extracellular matrix in exploring the progress made in computational fluid dynamics simulation to study the mechanism of nanoparticle delivery and interactions with cancerous tumors. The convergence of Machine Learning algorithms and CFD simulation in tumor-nanodrug interactions. The application of ML algorithms provides high predictive accuracy of nanodrug delivery that can benefit cancer biomedicine research by predicting how flow affects drug efficacy. The future of the ML-CFD is detailed to include imaging and 3D-CFD simulations to increase the credibility of these models and advancement to translational clinical research. This review concluded by urging collaborative efforts for a multiscale approach by biomedical engineers and scientists, as well as oncologists, to develop a modeling framework that advances precision medical care for effective cancer treatment. Standardization of the model and approaches, together with nanoparticle synthesis, is recommended to advance this research to the translational and clinical stage.

## Graphical Abstract

**Figure:**
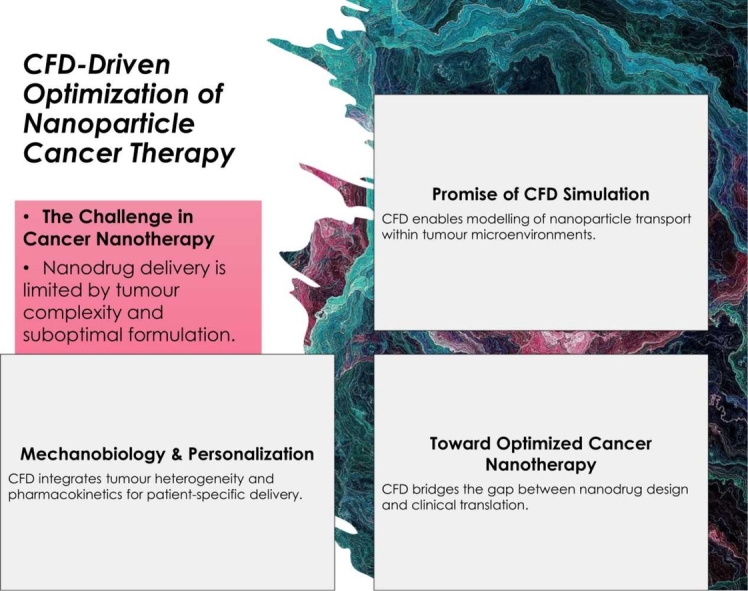

## 1 Introduction

Aberrant cell growth, angiogenesis, and metastasis are among the many cancer pathologies that necessitate the development of effective therapies [1]. In the United States of America number of people living with a cancer diagnosis is anticipated to exceed 22 million by 2035 [2]. According to the World Health Organization, new cancer cases are expected to exceed 20 % in the next decade, with approximately 70 % of cancer-related deaths recorded in low- and middle-income countries., This highlights the essence of addressing the development of effective therapy measures [2]. In the existence of conventional cancer treatments, including surgery, chemotherapy, radiotherapy, targeted therapy, precision, and tailored therapies [2], [3], 19 million new cancer cases are still reported annually worldwide, with nearly half of these causing cancer-related deaths [2], [3], [4], [5]. Copious newly discovered cancer drugs fail clinical trials, making the process of cancer therapy discovery and development an expensive, complex, and time-consuming process [6]. Despite the progress made in imaging, surgery, radiotherapy, and chemotherapy, the pursuit of a universal cure for cancer may remain a conceptual misapprehension. Approved drugs and treatment strategies often have debilitating off-target effects and limited efficacy, especially in the advanced/later stages of cancer [4], [7].

**Fig. 1:**
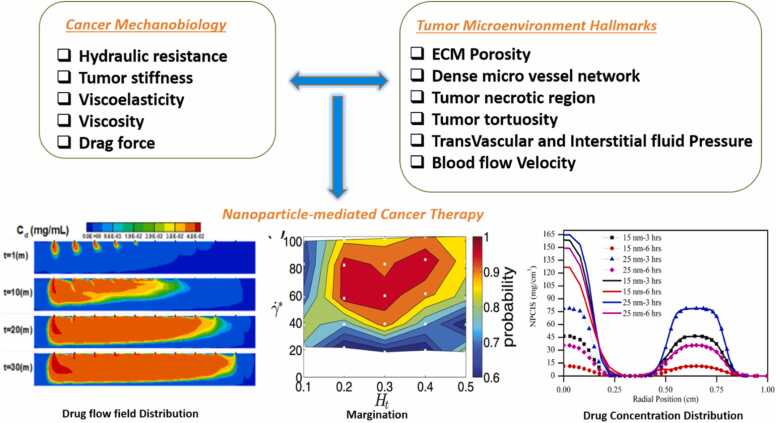
Tumor growth induced mechanical factors together with TME biological hallmarks, which influence the drug-tumor interaction [20], [25], [26], [27], [28].

An obvious constraint of chemotherapeutic drugs is the failure to reach a targeted area without off-target effects. Cancer biology explores intricate interactions between the aberrant cellular domain and the complex orchestration of multicellular systems [8]. Contingent on tissues of origin, cancer cells are identified as benign or malignant tumor cells; these cells exhibit a covert capacity to spread to healthy tissues, raising serious concerns with regard to the paradoxical use of physiological processes [4], [9]. From an engineering perspective, two key considerations to be made in developing effective cancer treatment are drug transport and drug conversion [10], [11] in the tumour microenvironment (TME). Cancer mechanobiology and cancer biological hall marks influence the biophysical variables that have a significant effect on effective drug delivery [12]. The growth-induced mechanical forces that the tumor exerts on the surrounding tissues alter the vasculature's distribution and structure, elevates interstitial fluid pressure, and interferes with delivery efficacy (Fig. 1). These effects occur on a variety of length and temporal scales where convection-dominated transport of larger macromolecules in the extracellular matrix is being contrasted with the rapid diffusion of single molecule agents like cytotoxic agents [13]. Among several therapeutic approaches used to combat cancer, cancer fluid mechanics and mechanobiology emerge as a pivotal factor in creating targeted cancer treatment.

Blood, interstitial fluid (IF), and lymphatic fluid have distinct biophysical properties, it is therefore simple to distinguish the mechanical stresses exerted on tumor tissues during drug transport and delivery by each of these factors [14], [15]. Atypical blood and lymphatic properties promote tumor development. While the lymphatic fluid is laminar, sluggish, pulsatile, and driven by viscosity [14], [16], the beating of the heart stimulates flow and circulation, where flow in arteries is turbulent but laminar [14]. Interstitial fluid pressure (IFP) gradient is elevated by tumorous blood vessels that are hyperpermeable and leaky [14], [17]. Brisk growth of the tumor causes solid stress, which constricts the cancer-associated vasculature and increases IFP gradients in the tumor tissue [14], [18]. Interstitial fluid pressure is robust in the tumor core but diminishes as it travels outward [14]. Computational fluid dynamics models IFP changes within the tumor and depict how it contributes to an increase in fluid convection and diffusion [14].

### 1.1 Fluid mechanics integration into cancer biology

The cancer fluid system is modeled by adopting a set of equations that describe blood flow, interstitial transport, and their interaction throughout the tumor. Usually, central assumptions are made on the blood and the interstitial flow, the pressure differences for fluid exchange, and the spatially resolved information of tumor tissues extracted from clinical images or literature [19]. For these models, boundary conditions are set to showcase flux and pressure continuity at junction points of the vascular network and within the tissue region. Blood flow through a network of vessels is described by Poiseuille’s law, which relates flow to channel radius, pressure, and blood viscosity. Transfer across the vascular network is described by [20] by relating extravasation rate to vessel permeability and the pressure difference between the vessel and the tissue. Velocity and direction of interstitial flow are established through Darcy’s law, which relates these variables to the pressure gradient and the hydraulic conductivity of the tissue [14].

Fluid mechanics and biological mechanisms of the tumors can be used as a point of focus to develop effective therapy. Drug movement through blood vessels and the tumor microenvironment is a major focus because with limited drug delivery the tumor grows, and cancer cells become resistant to treatment [21]. Barriers to drug delivery include deformed blood vessel walls, irregular tumor shapes and the complicated tumor chemistry [17], [22], [23]. While factors affect drug penetration and perfusion into the tumor, most drugs are diffusive due to the low-pressure gradients near the tumor edges caused by non-functional lymphatics [14]. Tumors compress lymphatic and blood vessels, subsequently interfering with drug transport [14], [24]. All the drugs that enter the tumor tissue will not always reach the cancer cells because some of them may be lost through binding to the extracellular matrix or reabsorbed by the tumor microvasculature. Moreover, quantity of free drugs accessible for more profound tissue penetration is restricted [25]. To maximise treatment, it is essential to deliver sufficient anticancer therapy to tumor sites. Nevertheless, it is still difficult to observe drug transport and investigate drug's spatial distribution in the tumor microenvironment [26]

### 1.2 Fluid simulation of nanoparticles to cancer tumors

Nanomedicine has been adopted into cancer biomedical research because of the small area-to-volume ratio and its quantum size. These features enhance the precision of nanoparticles in targeting tumors for therapy and diagnosis. The potential to reduce side effects and low dosing frequency of nanodrugs is a promising field of research for localized and targeted drug delivery of cancer therapy [29]. Nanodrug delivery systems have demonstrated benefits in the treatment of cancer through improved pharmacokinetics, accurate targeting of tumor cells, fewer adverse side effects, and decreased drug resistance [30], [31]. Nanotechnology applied to drug delivery for cancer treatment has been shown to play a significant role in overcoming drug resistance [30]. Effective nano-based therapy largely depends on the enhanced permeability retention effect (EPR) and the active targeting advantage of nanoparticles [32], [33]. Nanoparticles reach and spread across the tumor through the vascular network of the circulatory system [21]. Nanoparticles proceed to cross into the tumor interstitial matrix and to the cancer cells [34]. The abnormal tumor microenvironment and the altered homeostasis are key contributors to the complexities of drug delivery to cancer cells [35], [36], [37]. It is therefore impossible for drugs to uniformly and effectively reach tumors, which results in inadequate local drug concentrations and failed cancer treatment. The heterogenous drug distribution within the tumor is the key reason for deficient therapeutic effect of nanoparticles as cancer therapy [38].

There are three sequential steps involved in drug transport through the tumors; (1) transport via capillary walls in the extracellular matrix, (2) transport to the tumor cells via the extracellular matrix and (3) transport through tumor cell membrane into cancer cells [39]. Since most drugs encounter transport restrictions, there is high pertinence for extensive research that investigate drug transport across the tumor matrix [39], [40]. Computational modeling is adopted in cancer biomedicine research to understand the influence of fluid mechanics on mechanisms of cancer progression and treatment. Physiological obstacles (irregularly shaped microvasculature, the elevated solid stress or compression of micro vessels) and the very dense extracellular matrix of the tumor microenvironment, elevated interstitial fluid pressure as well as hydraulic conductivity, make drug delivery and treatment complex and case specific. CFD Simulation research focuses on predicting drug delivery in cancer cells [41], [42] and uncovers effects of varied tissue transport properties on drug perfusion and penetration [43].

Jain and Kiseliovas [44], [45] highlighted that blood and interstitial fluid flow computation is essential for analysis of anticancer drug transport. This approach helps to build facile drug models with high simulation efficiency of fluid flow [46]. In theory, computational simulation techniques can be adopted to evaluate motion of drug loaded NPs as this would help understand the impact of flow on aggregation and distribution patterns of nanodrugs [46]. Development of CFD simulations that can correctly predict drug delivery and tumour treatment; improvement and optimization of those treatments to provide new therapies can help personalise treatment. Computational fluid dynamics enables researchers to conduct numerical and mechanistic experiments that reveal a detailed flow and drug distribution information [26]. Literature has majorly established that tumor initiation, growth and invasion is tightly couple to blood supply characteristics [47]. Jain and other authors [11], [35], [48], [49] added that interstitial flow within the tumor microenvironment controls access to nutrition and oxygen. Therefore, both blood and interstitial flow are key players in the heterogenous delivery of cancer drugs, causing the varied treatment response in patients [19]. There have been some efforts to adopt CFD in characterising tumor related flow and the effect it has on enhancing cancer treatment. Developing active targeting nanodrugs has proven to be a challenge in cancer clinical research [6], with the cause being the drug and target interaction. Selecting target molecule and drug design limits the discovery and efficacy of functional nanodrugs [21], it is therefore a necessity to carry out in-depth research on nanoparticle-tumor interactions.

As evident in literature, there exist complexities in developing effective nanoparticle-based therapy, generally due to tumor microenvironment heterogeneity and lack of detailed understanding of the mechanisms of nanoparticle transport in the tumor. Tackling various drug-delivery challenges is a complex issue of which its resolution requires interdisciplinary effort from *in vitro, in vivo,* and realistic numerical simulations. Therefore, the aim of this review is to better understand the efforts of computational fluid dynamic simulations in nanoparticles delivery to tumors. In this review we aim to answer the following questions:

• To what extent have the biophysical characteristics of nanoparticles been simulated to target tumors and what are the factors that hinder NP preparation as cancer treatment?
• What tumor characteristics and drug parameters have been investigated with simulations when targeting nanoparticles?
• What CFD simulation and tumor reconstruction approaches have been used to study drug delivery to tumors?
• How has ML integration improved fluid simulation of nanodrug delivery to tumors?

In the remainder of this review study, methodology will be followed results, discussion, and conclusion.

## 2 Materials and methods

The systematic review followed the general principles outlined in the Center for Reviews and Dissemination (CRD) guidance for conducting reviews in health care. The systematic review is reported in accordance with the Preferred Reporting Items for Systematic Review and Meta-Analyses (PRISMA).

### 2.1 Data source and search strategy

A literature search to identify original papers on IEEE, PubMed, and Science direct was conducted. Article search was conducted with following keywords (((Computational fluid dynamics simulation) AND (cancer) AND (nanoparticles) AND (drug delivery))) with the year filter of 2014–2024. For IEEE and PubMed search review and magazine filter was included in the article search. The initial search was in October 2024, updated in March 2025 and the final one on October 2025 to include 2025 studies.

### 2.2 Study selection and data collection

A two-stage process was adopted to assess the downloaded papers for inclusion in this study. Firstly, all titles and abstracts from the electronic database search were screened to identify the potentially relevant articles to be retrieved. Following this full text copies of the studies were obtained and assessed for inclusion using eligibility criteria outlined in Table 1 below. Any disagreement was resolved through discussion stage and if necessary, consult with a third reviewer.

**Table 1:** Shows selection criteria for study eligibility.

**Inclusion Criteria**	**Exclusion Criteria**
Cancer/tumors	Not Cancer
Nanoparticles	Not Nanoparticles
Fluid simulation	No fluid Simulation
Original experimental paper, conference paper/proceedings, Literature review	Book/Book chapter, magazine,

### 2.3 Limitations of Data Collection and Study Selection

Reliability and quality of the literature review maybe affected by some challenges faced during the initial state of data collection and screening. Relevant studies unavailability to UNISA’s library or with paywalls were omitted. Using limited keywords to such through selected databases results in a search strategy bias that could have resulted in missing relevant studies. Only positive results are reported in detail in majority of published studies, these skews the available data.

During study selection personal judgement of studies can introduce subjectivity. Inclusion of studies with poor methodology and those that are not comprehensive contributed to weak conclusions during review. Heterogeneity of study designs and results among the studies make the study comparison difficult and biased. Some studies are extensive, therefore including multiple entries from form the same study have a potential of distorting the findings. Some fundamental concepts were established back in the years and caried through studies into current research. Reporting on these concepts and citing older literature gives the impression of reporting outdated conclusions.

## 3 Results

### 3.1 Data extraction and statistical analysis

**Fig. 2:**
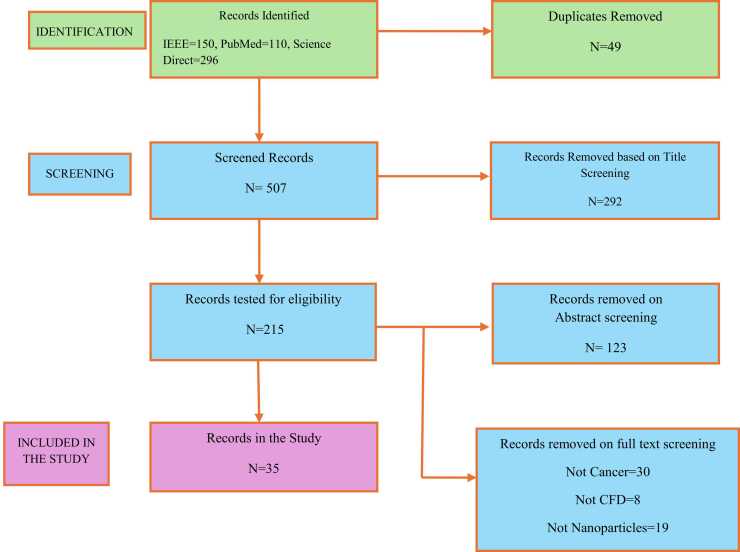
A schematic depiction of the workflow adopted in collecting and screening relevant studies to include in the review as per selection criteria explained in methodology.

Data collected included year of publication, type of nanodrug, type of cancer and CFD simulation models. Herein, data extraction was performed by one reviewer and checked for accuracy by two other reviewers. The screening process followed the flow diagram in Fig. 2 below. References of the study were exported from RefWorks citation manager as a BibTex file for bibliometric analysis. The analysis was performed on R-studio version *2024.12.0.467* by using the bibliometrix package. Microsoft excel was adopted to compute frequency distributions for nanoparticles, tumor and models used in the studies.

Different nanoparticles and their tumor interaction behaviors are investigated by researchers as summarized in Table 2. These studies mainly investigated spherical nanoparticles ranging from 5 nm to 2000 nm, the nanoparticles are either conjugates, cancer drug encapsulations or inorganic as shown in Table 2. Several of these studies did not specify the tumor type that was studied, however brain and related, liver, pancreatic, hepatic, melanoma, breast cancers were investigated.

**Table 2:** Nanoparticle and type of cancer used in the studies.

**Nanoparticle type**	**Size and Shape**	**Tumor type**	**Simulation Model**	**References**
Iron oxide	cubes	Brain (Glioblastoma)	3D Multiphase Porous-Media Tumor	[50]
Magnetic	Generic	Generic solid tumor	Generalized 2D Numerical-machine Learning	[51]
Superparamagnetic Iron Oxide	Generic	Generic solid tumor	Generalized 2D Hybrid CFD-AI	[46]
Magnetic	Generic	Generic Solid Tumor	2D CFD^1^ Levenberg-Marquardt Neural Network	[52]
Hybrid magnetic (Titanium-alloy-Gold mixture, Alumina and Gold, Copper-SWCNT)	Generic	Generic solid Tumor	2D Hybrid Mathematical-Machine Learning	[53]
Magnetic	Generic	Generic solid tumor	2D Hybrid ML-CFD	[54]
Magnetite	Spherical, 15 nm	Brain (Glioblastoma multiforme)	2D Computational Bioheat Model	[55]
Gold-coated iron oxide magnetic	15 nm	Skin	3D CFD	[56]
Liposomal-encapsulated MZ1	100 nm	Breast	3D Multiphysics Simulation	[57]
Magnetic	Generic	Generic Vascular	2D Levenberg-Marquardt Neural Network	[52]
Generic	Spherical and Ellipsoidal, 100nm−3.84 µm	Vascular	2D and 3D Mesoscopic Hydrodynamic Simulation	Muller, 2014
Generic	Generic	Brain (Glioblastoma)	3D Multiphysics Simulation	[58]
Doxorubicin-polymer conjugate	Spherical, 100 nm	Generic Deep-lying tumor	3D Mathematical Model	[59]
Gold	Quasi-spherical, 22 nm	Breast	3D Microscale CFD-DEM-DLVO^2^ Framework	[60]
Doxorubicin conjugate		Generic solid tumor	2D Multiscale Mathematical	[61]
Liposome-encapsulated Doxorubicin	Spherical, 100 nm	Brain (Malignant glioblastoma)	3D Multiphysics Mathematical Model	[62]
Liposomal-encapsulated Doxorubicin	Spherical	Brain (Glioblastoma multiforme)	3D Mathematical Model	[63]
Thermosensitive Liposome-encapsulated Doxorubicin	Spherical	Liver	3D Numerical Simulation	[64]
Generic Magnetic drug carrier	Spherical, 200 nm	Generic Solid tumor	2D and 3D Mathematical Model	[38]
Carboxylate-modified polystyrene fluorescent latex	Spherical, 500–2000 nm	Generic Metastatic tumor	3D CFD model	[44]
Generic	1,10 and 50 nm	Breast solid tumor (Mammary carcinoma)	3D Dynamic-tissue modeling Framework	[13]
FITC-Dextran	Spherical, 10 nm	Generic Solid tumor	2D Eulerian-Eulerian two-phase mixture model	[26]
Liposomal Doxorubicin	Spherical, Generic	Vascularized squamous cell carcinoma	2D Multi-scale Computational Model	[65]
Superparamagnetic Iron Oxide encapsulated Mitoxantrone	Spherical, 50 nm	Melanoma	3D Multiphase Porous-Media Model	[66]
Aluminum oxide	Spherical, 200 nm	Pancreatic and Hepatic	2D CFD simulation	[67]
Generic	Spherical, 10, 20, 50, 70, 80, 100, 160 nm	Vascular	3D Agent-Based Model using FLAME^3^ Framework	[68]
Magnetic	Spherical, 10 and 100 nm	Brain (Glioblastoma multiforme)	3D CFD-DEM Model	[69]
Aptamers	Linear single strand nucleic acid	Brain (Glioblastoma multiforme)	2D CFD simulation	[70]
Generic nanofluid	Spherical 1–100 nm	Generic Vascular	2D Numerical Simulation	[71]
Generic Newtonian nanofluid	Spherical, 10 nm	Generic tumor surrounding blood vessel	2D Mathematical Model	[72]
Thermosensitive Liposomes	Spherical	Generic Solid tumor	Numerical 3D Porous-Media Model	[73]
Copper oxide and Silver	Spherical, 120 and 40 nm	Tumor angiogenic vessels	3D CFD Simulation	[74]
Generic	Spherical, 25 nm	Pancreatic ductal adenocarcinoma	2D Multi-scale and Agent-Based Model	[75]
Magnetic	Spherical, 10 nm	Generic Porous Tumor	2D Multiscale Model	[76]
Magnetic Nanoparticles	Spherical	Generic necrotic Solid tumor	2D Mathematical Model	[77]

1Computational Fluid Dynamics; 2 Discrete Element Model- Derjaguin–Landau–Verwey–Overbeek 3 Flexible Large-scale Agent Modeling Environment

**Fig. 3:**
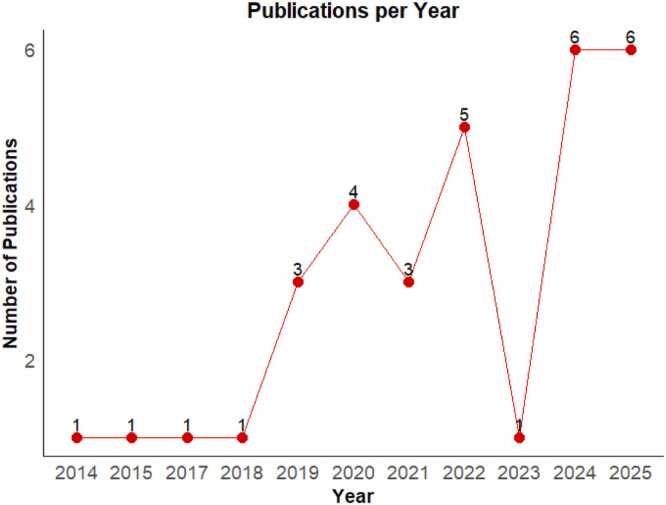
Article production for each year from 2014 to 2025.

There is a steady low output from 2014 to 2018 with 1 publication recorded each year. Significant growth starts in 2019, peaks in 2022 and drops in 2023. A strong recovery and highest output is observed for the years 2024 and 2025.

**Fig. 4:**
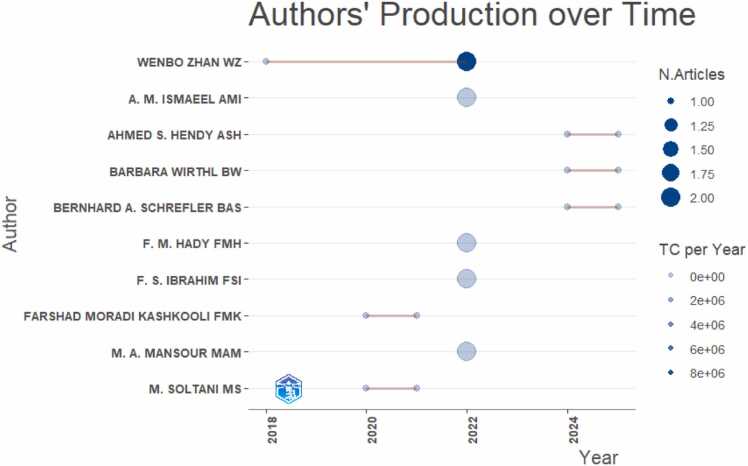
Number of documents each author has published across the years 2014–2025 along with their citation impact.

**Fig. 5:**
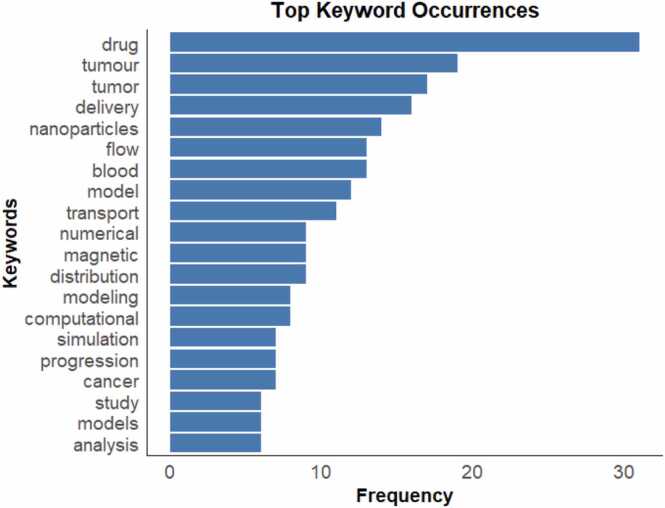
A horizontal bar plot showing the most frequently used keywords in the analyzed studies.

This graph shows a timeline of publications and the authors over the years. Each dot represents an author’s publication in a specific year and the size of the dot corresponds to the number of articles published that year. The larger dots represent more articles. The horizontal line connects the years in which the author published, showing their activity span. The color intensity indicates Total Citations per year where darker dots imply a higher citation impact (Fig. 5)

“Drug” is the most frequent keyword, appearing about 30 times, suggesting a strong research focus on drug-related topics. “Tumor” and “Tumor” both frequently, approximately 20 times each, indicating that the studies are related to cancer, which it true to the aim of the review.

**Fig. 6:**
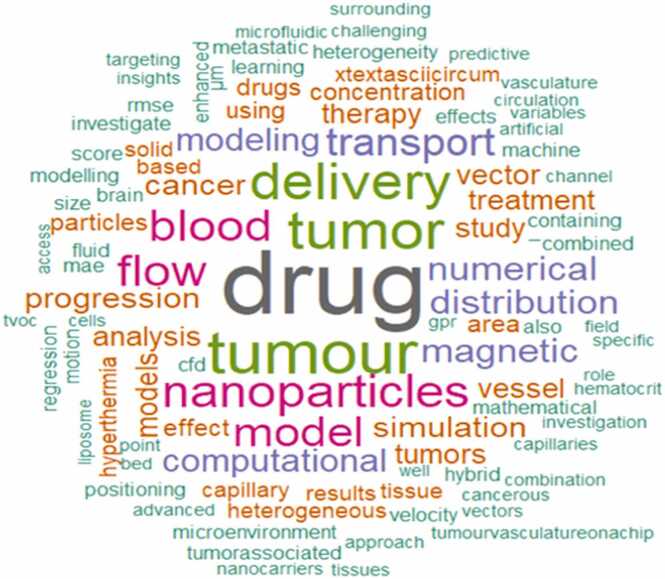
Word-cloud showing key term occurrences in the studies.

Fig. 6 shows the word cloud of keywords in the studies. Largest words (drug, tumor, tumor, delivery, flow, blood, nanoparticles, model, simulation) show most frequent keywords. Medium sized words (therapy, computational, numerical, and magnetic). Smaller words (hyperthermia, heterogenous, capillary, microenvironment) represent a niche or emerging topics.

**Fig. 7:**
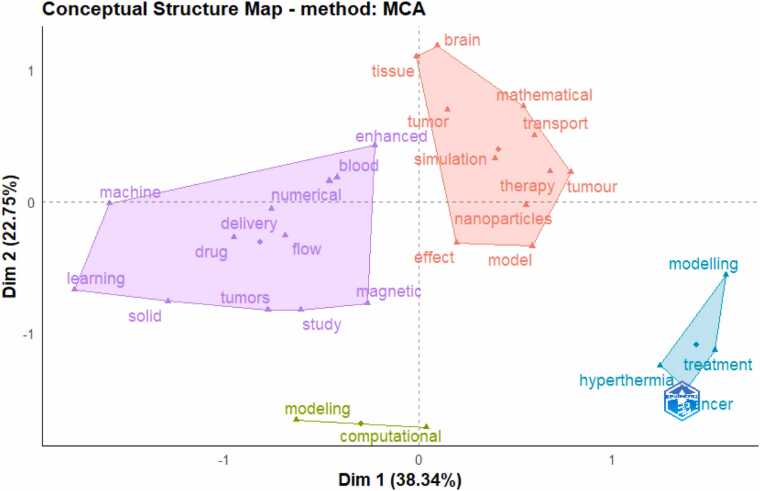
Visualization of thematic clusters of keywords in the studies.

Fig. 7 shows a Conceptual Structure Map created using Multiple Correspondence Analysis (MCA) in bibliometric analysis. The keywords are based on their co-occurrence in the dataset to help identify the major themes and how they relate to each other. The clusters show groups of keywords that frequently appear together; the closer the keywords are on the map, the more related they are conceptually. Larger clusters indicate broader themes, while smaller clusters indicate niche topics.

**Fig. 8:**
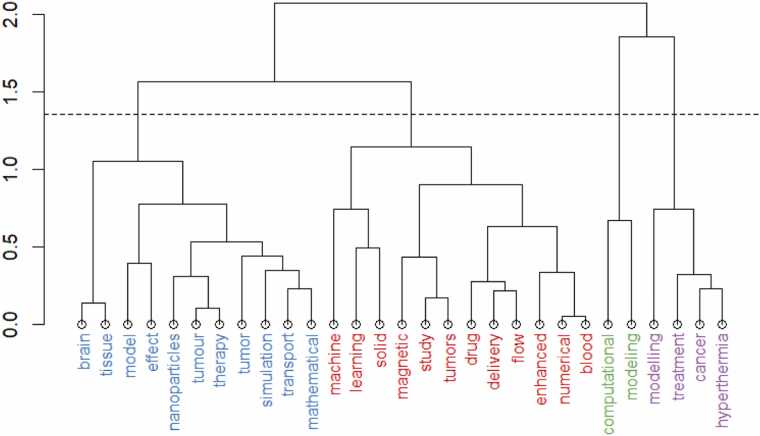
Hierarchical clustering dendrogram of keywords.

Fig. 8 is a display of keywords commonly used in bibliometric analysis to show conceptual relationships between terms. The dendrogram groups words based on their similarity or co-occurrence in the dataset. The bottom (leaves) shows individual keywords as described from the dataset. Branches show how keywords cluster together into different thematic groups that are represented by color. Shorter branches show stronger similarity between keywords, while longer branches show weaker similarity.

**Fig. 9:**
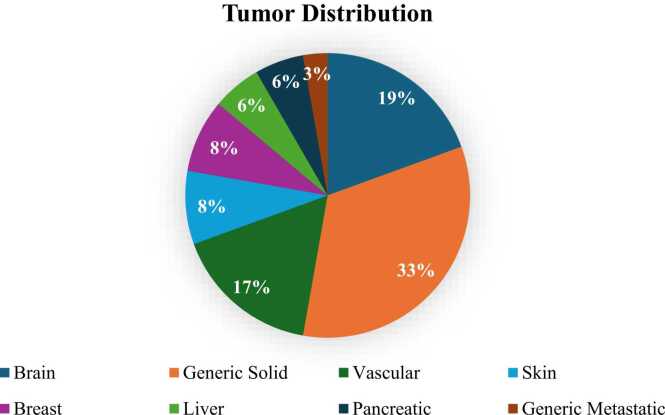
Frequency of tumor types in the CFD models of the collected studies.

Tumor distribution (Fig. 9) shows that 53 % is attributed to unspecified cancer, with generalized solid tumors having the highest frequency (33 %), followed by vascular tumors (17 %) and finally metastatic tumors (3 %). There are only five tissue-specific cancers studied, namely: brain, breast, pancreatic, and skin tumors.

**Fig. 10:**
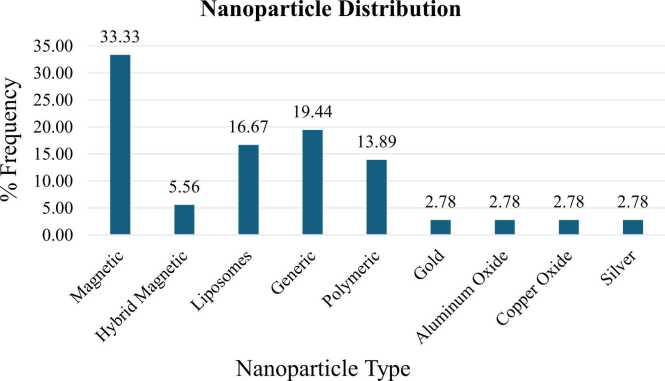
Distribution of nanoparticle types adopted as cancer nanodrugs in this study.

Magnetic nanoparticles are the most studied nanoparticles as cancer nanodrugs, with a distribution of 33 % (Fig. 10). Liposomes and polymeric nanoparticles make up 16.67 % and 13.89 % while generalized nanoparticle make up 19.44 % of the distribution. Finally, metallic nanoparticles in these studies contribute 2.78 % to the distribution respectively.

**Fig. 11:**
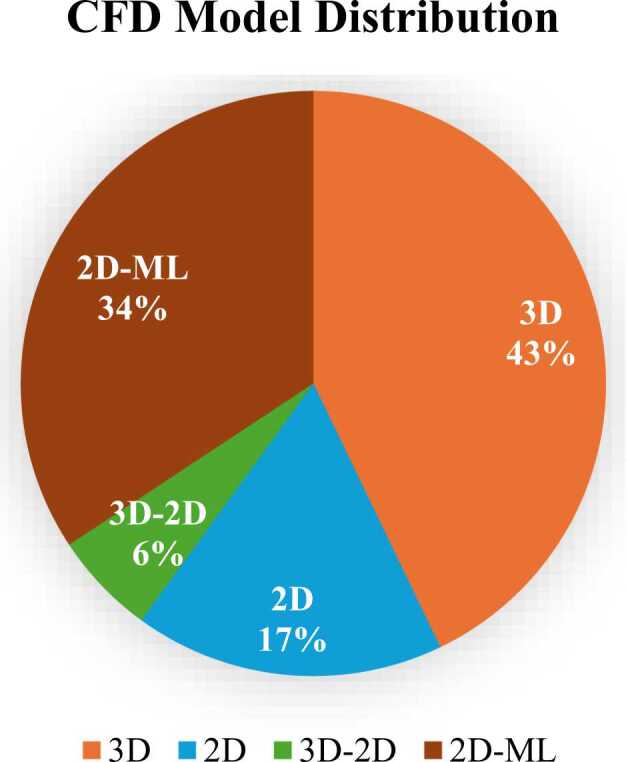
It shows the percentage breakdown of different types of Computational Fluid Dynamics (CFD) models.

As shown in Fig. 11, the chart indicates that 3D CFD models dominate (43 %), followed by 2D models combined with machine learning (34 %), while pure 2D models (17 %) and hybrid 3D-2D models (6 %) are less common.

#### 3.1.1 Nanoparticle physicochemical properties and their interaction with the Tumor microenvironment

**Fig. 12:**
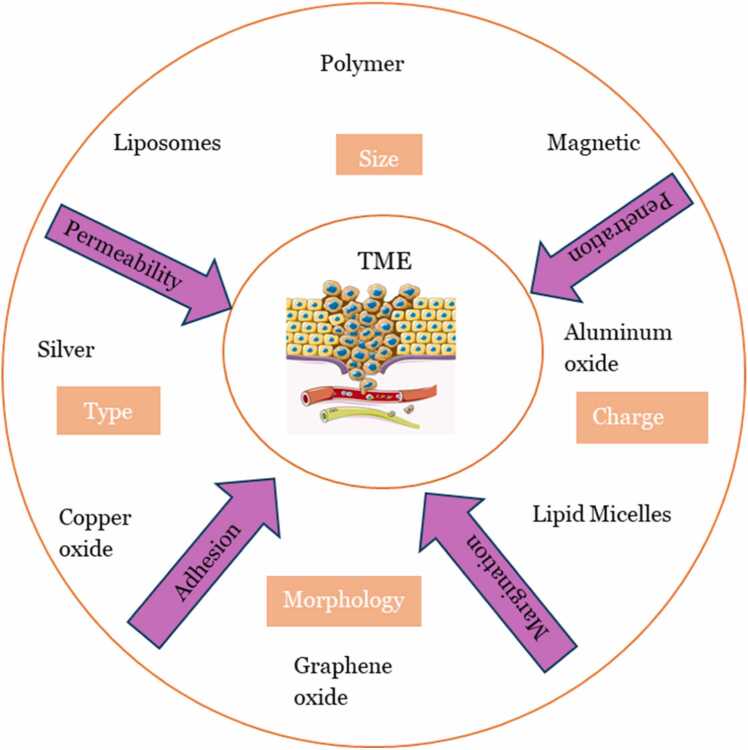
Nanoparticle effectiveness in treating cancer is dependent on physicochemical properties, which influence the interaction with the complex tumor microenvironment.

Developments in the field of nanomedicine continue to break new echelons in cancer treatment, diagnosis, and regenerative medicine [78], [79]. Nanomedicine-based cancer therapies are advancing, but the efficacy is hampered by limited knowledge of drug delivery dynamics that are affected by nanoparticle physicochemical characteristics and properties of the tumor microenvironment (Fig. 5). Studies have shown that nanodrugs have better bioavailability and biodistribution than free drugs and thus confer enhanced pharmacokinetic and pharmacodynamic effects to the patient [80], [81] (Fig. 12).

Drugs conjugated to nanoparticles are advantageous in increasing the therapeutic window as compared to free drugs. In comparison of free and nano drug, [62] showed that liposome-encapsulated doxorubicin coupled with convection-enhanced delivery (CED) enhances therapeutic outcome in the brain tumor. This is owed to the slow-release rate that reduces drug elimination and improves distribution. The study showed that CED-mediated delivery of liposomal doxorubicin also penetrates distal tumor regions as compared to free doxorubicin, as observed by [82] While this study offers valuable insight into CED optimization using liposomal doxorubicin and sets a strong foundation for future experimental and translational research, there are improvements to be made. Assessing the model with different nanodrugs and/or multi-drug regimens that have different behavior under CED will expand its drug applicability. Cytotoxicity of the nanodrug is not evaluated thoroughly; endocytosis and intracellular drug dynamics can be included to simulate cell death, drug internalization and efficacy more realistically. Endocytosis and intracellular drug dynamics are excluded, although these are critical for doxorubicin’s cytotoxicity. Brain tumors are heterogenous and anisotropic, therefore assumptions of isotropic homogeneity are not realistic.

Nordling-David and team [82] addressed poor drug delivery across the BBB, a critical challenge in glioblastoma multiforme treatment. Liposomal Temozolomide (TMZ) reduces edema, a relevant symptom in the treatment of GBM. *In vitro* evaluation of liposomal TMZ showed both a concentration- and time-dependent decrease in cell viability. Therapeutic evaluation *In vivo* analysis revealed that 70 % of rats treated with TMZ survive as compared to 25 % treated with saline. While the liposomal TMZ proposes a novel formulation tor CED, the therapeutic advantage over free TMZ delivery is marginal and statistically insignificant (P = 0.053) when taking survival and tumor volume reduction into consideration. This weakens the argument of its superiority. Brain biodistribution of PEGylated Gd-DTPA liposomes evaluated in intracranial glioma rat model by MRI showed enhanced images following CED. Co-encapsulation of TMZ with an MRI marker was attempted to explore the dual therapeutic and imaging capabilities of the liposomal TMZ. However, Gadolinium diethylenetriaminepenta-acetic acid (Gd-DTPA) failed because removal of free un-encapsulated TMZ reduced concentration of liposomal TMZ. These limits simultaneous therapeutic and imaging capabilities. This should not discourage further research but deeper exploration of potential dose optimization and combination therapies. In future, histological confirmation of drug localization and toxicity can be included to strengthen claims about liposomal TMZ safety and efficacy.

The therapeutic activity of cancer nanodrugs depends on the type of nanoparticles. For instance, Mulpuru and co-workers [74] showed that while the NPs have selective toxicity to tumorous than normal capillaries, silver and copper oxide nanoparticles have different retention times. Magnetic nanoparticles achieve more specific tumor targeting and enhanced uptake while reducing systemic toxicity, due to magnetism [83], [84]. Even though magnetic nanoparticles can be directed to the target tissue by external magnetic field, the magnetic forces exerted on the nanoparticles fall off rapidly with distance, making tumor targeting a challenge. To address this, a computational model adopted a continuum approach to model nanoparticle transport based on diffusion-advection equations in a multiphase porous media. It was revealed that at the edge of the magnet where magnetic force is weak, it is hard to capture nanoparticles leading to limited tumor spheroid penetration [66]. An integration of multiphase porous-media modeling with thermal dynamics is a novel and comprehensive approach but sole reliance on computational modeling and lack of validation with experimental data limits the applicability of the model. The comparison of lumped Pennes’ bioheat equation and discrete vascular models adds depth and realism to the simulation. While parameter sensitivity analysis is mentioned, it is not fully explored. A more rigorous global sensitivity would strengthen the conclusions. The heavy reliance on computational modeling with biological or clinical correlation affects the translational relevance of this model. An idealized spherical tumor with homogenous vasculature oversimplifies the realistic vascular tumors.

The interaction of nanoparticles with the tumor tissues is an important factor to be considered in precise delivery of nano chemotherapy to tumors. A team led by Xie [60]conducted *in vitro* experiments validated through CFD-DEM simulation to observe tissue penetration of gold nanoparticles (AuNPs) into tumor spheroids. The tumour tissue is constructed through SEM and 2-photon microscopy images of tumour spheroids and the drag model which accounts for nanoparticle-fluid interaction was adopted to quantify AuNP transport through the extracellular pathway. DLVO (Derjaguin, Landau, Verwey, and Overbeek) sub-model is implemented to consider *Van der Waals* and repulsive electrostatic forces involved in AuNPs transport and tumor penetration. A combination of two-photon and scanning electron microscopy with a 3D CFD-DEM simulation model enhances reliability and depth of findings, while the use of DVLO sub-model adds realism to the particle interaction modeling. Their findings revealed that gold nanoparticles penetrate the tumor and attach (60 % intratumoral NPs attach to cells and 40 % to the extracellular matrix) through the extracellular pathway. Nanoparticle uptake and aggregation in the tumor are dependent on nanoparticle concentration and tumor density. Both the simulation and experiment showed a relatively random nanoparticle distribution across the tumor tissue. Aggregates of nanoparticles can be observed for both cases, and the penetration curve tendencies are similar for both the experiment and the simulations. Inhibiting energy-dependent intracellular uptake enables the study to isolate and analyze the extracellular penetration mechanism effectively. This provides a crucial understanding of passive transport dynamics in tumor tissues. Focusing on four different NPs' motion modes (direct penetration, initial uptake, reactivation, and escape) provides a nuanced comprehension of NP behavior at a microscale level. This research paper is highly technical for broader biomedical or clinical readers or specialists. Simplifying or summarizing key equations and mechanisms in lay terms could improve readability and interpretability. The role of extracellular matrix (ECM) in trapping nanoparticles is well characterized, with implications for drug delivery efficiency. The ECM is modeled as densely packed spherical particles, ignoring the large collection of distinct biochemically relevant components that exists in the interstices of cells as many spherical and fiber-like structures. While this is justified for computational feasibility, the simplification overlooks important structural influences on penetration. Future work should explore a hybrid ECM geometry and validate these assumptions with experimental work. The assumption of uniform IFP does not reflect real tumor heterogeneity.

Margination and adhesion are important biophysical properties in tumor and nanoparticle interaction that guides particle penetration. In a study by Muller and coworkers [28], simulation studies revealed that micron-sized ellipsoidal particles are more favorable to drug delivery than the elliptical nanoparticles due to their excellent adhesion properties and slower rotational dynamics. In this study blood was modelled as a suspension of red blood cells and nanoparticles, with blood flow computed through 2D and 3D simulations. The simulation revealed that the larger the hematocrit the better the nanoparticle margination because of increase in available space for the nanoparticles. Larger nanoparticles are highly distributed next to the blood vessel wall due to their interaction with red blood cells while smaller nanoparticles are uniformly distributed and highly concentrated in the vessel center. Larger nanoparticles showed a high adhesion potential at both high and low hematocrit levels. The size and shape of nanoparticles is explored to greater lengths to investigate their behavior within the tumor. Moreover, [28] found through 3D simulations that larger spherical nanoparticles have better margination and showed higher adhesion capacity to blood vessel walls as compared to ellipsoidal nanoparticles. The 3D simulations in this study showed that a sphere is subject to uniform rotation while ellipsoids display tumbling dynamics. Angular velocities of margination revealed that ellipsoidal particles rotate slower within the red blood cell free layer than spherical carriers. While the study systematically investigates the effects of particle size, shape, and vessel diameter on margination there are other limitations to be addressed. The overreliance on simulations without *in vivo* or *in vitro* validation minimizes the credibility of the model. There is also limited biological contest because the study heavily focuses on physical parameters but does not explore biological factors such as endothelial interactions, adhesion mechanisms and immune responses. Future research should include adhesion and margination dynamics, receptor-ligand interactions, and cellular uptake. The study acknowledges that margination is a prerequisite but not equivalent to adhesion, yet it does not simulate adhesion directly. Future expansion of the study should explore non-spherical shapes beyond the ellipsoid, such as rods, which may have unique margination and adhesion profiles. A consideration of pathological conditions where blood flow and vessel properties differ significantly.

In a previous study, [68] adopted an agent-based FLAME framework to model nanoparticle behavior within capillaries in the presence of red blood cells to reveal that nanoparticle delivery is reduced in polydisperse samples with 100 nm and 160 nm populations, because some nanoparticles are too large to pass through the tumor vessel fenestrations. Conversely, in polydisperse samples around 70 nm, there showed a slight increase in delivery to normal tissue, possibly because presence of smaller particles that can pass through the 60 nm cutoff. This showed that there is a marked difference in size between polydisperse nanoparticle uptake into tumor fenestrations and those in the vessel with smaller sizes being taken up more readily. The adoption of FLAME framework allows for scalable and parallel simulations to enhance computational efficiency the integration of agent-based modeling with CFD. Incorporation of red blood cell dynamics is often neglected in simpler models but are crucial to realistic blood flow simulation. In this paper, tumor targeting via the EPR and exploration of polydispersity. These are realistic concepts that guide therapeutic activity and efficacy of nanomedicine. Nanoparticles are assumed to be inert and non-interacting, which may not hold true for all formulations, especially those with active targeting ligands or surface charges. Future studies can include NP interaction with protein corona which has been proven to drastically alter nanoparticle interaction. RBCs are treated as rigid agents with a simple deformation behavior. Experimental validation of key findings, especially the predicted optimal nanoparticle sizes and dispersion patterns is recommended to establish credibility of the model. Tumor vasculature is modeled is primarily via fenestration size, but other features like irregular flow, leaky junctions, and hypoxia-induced changes are not considered. The study can be improved by incorporating deformable RBC models using immersed boundary or lattice Boltzmann methods for more accurate flow dynamics. The model can be extended the model to include active targeting mechanisms, immune interactions, and TME heterogeneity. Consider multi-scale modeling to bridge capillary-level dynamics with tissue-level delivery outcomes. While computationally efficient, the assumptions in the model overlook critical biomechanical interactions. The model is entirely in silico, experimental validation would strengthen the conclusion.

A study led by Gkountas [69] resolved Navier-Stokes equations into a 3D CFD-DEM model to compare 10 nm and 100 nm magnetic nanoparticles motion and interaction of MNPs in interstitial fluid flow. Magnetic field increases the number of MNPs passing through the MNPs, the model slightly overpredicts permeability in the absence of a magnetic field. The experimental data showed that without magnetic field, 2–6 % MNPs pass through the blood brain barrier (BBB) and 7–12 % for numerical simulation. In the presence of a magnetic field, 45 % passed through from experimental data and 46 % of permeability is recorded for numerical simulation. When exposed to magnetic field. In the simulation, a 30 % enhancement of 100 nm and 1 % of 10 nm MNPS passing the BBB is observed under a magnetic field. Magnetic field influences larger NPs navigation. Small nanoparticles do not obey the magnetic field, no potential aggregations can be formed, and finally the nanoparticle cannot withstand to the main flow of the blood. Therefore, the small MNPs are driven from the main flow and cannot cross the BBB. This implies that the magnetic force is very weak to navigate the MNPs to the desired direction. The magnetic field is treated as uniform and externally applied, but real-world applications may involve spatially varying fields and complex interactions with tissue. The model slightly overpredicts MNP permeability in the absence of magnetic fields compared to experimental data. Lower blood flow rates improve MNP accumulation near the vessel walls, enhancing their chances of crossing the BBB while high flow rates tend to carry particles away from the barrier before they can interact with it. The pressure gradient forced MNPs to move towards the endothelium. At initial velocity, 45 % of MNPs passed through, and an increase of 3 % was observed at half of the velocity. Applying a pressure gradient, 32 % of MNPs passed through. Therefore, blood velocity variation does not present a significant impact on MNPs crossing through the BBB compared to the existence of an external magnetic field. Through this model, it is demonstrated how the shape and spacing of endothelial cells impact how easily MNPs can pass through. Clearly, endothelial cell geometry affects transport because circular cells with larger intercellular gaps allow more particles to cross. The model assumes circular endothelial cells and periodic structures, which oversimplify the complex and heterogeneous nature of the BBB.

Wijeratne and co-workers [13] developed a fluid mechanics model with a compartment that simulated the effect of microvascular hemodynamics and IF flow on continuous drug delivery. The model used data from *n vivo* murine adenocarcinoma to simulate single-dose administration of cancer-killing drugs. In this model, nanoparticles cause tumor regression at both high and low tumor porosity as compared to larger nanoparticles that require high porosity to cause tumor regression. Despite the study taking aim to replicate popular EPR and porosity phenomena that guides drug efficacy there are limitations that deter the expansion of the study. All drugs are assumed to have identical pharmacokinetic properties that oversimplifies real world variability in absorption, metabolism, and clearance. Polydispersity of NPs and drug properties such as binding affinity and half-life enable a nuanced simulation of cytotoxic drugs against nanoparticles. Additionally, the study focuses on single-dose administration limits its applicability to clinical multi-dose regimens or combination therapies.

Yang and Zhan [58] adopted the mass conservation principle to govern nanoparticle concentration in brain tumor. This study evaluated how tissue hydraulic permeability influences CED of nanoparticle encapsulated doxorubicin, paclitaxel, temozolomide and carmustine. Their model showed that CED leads to a highly localized delivery outcome. This localized drug distribution is important in achieving precise delivery to minimize off target effects caused by high drug concentration in normal tissues. It was revealed that doxorubicin, a poorly absorbed drug, had the best retention and distribution. This drug also had low drainage in tumor tissues even though it had the lowest LD90 as compared to the other drugs. The study explores the underexplored contribution of tissue hydraulic permeability to convection enhanced delivery of multiple drugs. However crucial physicochemical properties of nanoparticles important in drug design are missing (Size, shape, material, and surface properties. A specific model should be developed for a particular type of nanoparticle together with in characteristics.

Akhtar and team [53] investigated the magnetic field effects’ dual functionality of magnetic nanoparticles in delivering drugs to tumors and reducing hydrogen peroxide levels in cancer cells. In a 2D CFD-ML hybrid model, nanoparticles were treated as passive carriers and their optimization compared through the Levenberg-Marquardt ML model. It was shown that higher Schmidt number predicted a wider dispersion of nanoparticles, and that an increase in magnetic field intensity aligns nanoparticle and blood flow due to increased shear stress. This in turn increased drug activation and reduced drug concentration at the tumor. This trade-off is crucial for balancing therapeutic effects and MNPs bioavailability in the tumor. The adoption of ML optimization of nanoparticle behavior modeled using the Levenberg-Marquardt neural network in this scenario predicted efficacy with high accuracy. Therefore, a combination of fluid dynamics magnetic control and machine learning improved targeting accuracy, drug delivery efficiency and reduced side effects paving way for development of personalized cancer therapies using smart nanoparticle systems.

In 2D FEM-CFD model designed by Maniotis [55], to assessed thermal interaction between magnetic nanoparticles and cancer tumors. Heat loss was determined through a numerical model that simulated NP behavior by the numerical integration of the Landau-Lifshitz-Gilbert (LLG) equation. This model revealed that when nanoparticles are exposed to an alternating magnetic field, they produce heat due to Néel and Brownian relaxation mechanisms. This heat raises the temperature of the tumor (41–45°C) to therapeutic levels hence killing cancer cells. The computational results complemented the experimental temperature values obtained from clinical studies using the same magnetic nanoparticle parameters. Despite the success of this study, the assumption of uniform MNPs dispersion does not reflect *in vivo* condition due to aggregation. Further research should focus on *in vivo* validation of the simulations. This should consider nanoparticle distribution, agglomeration effects, and non-uniform tissue properties to refine predictive.

Another 2D FEM model developed by Gas and team [56] applied a mathematical simulation approach for magnetic hyperthermia treatment of skin cancer. Magnetic field produced by gold coated MNPs was modeled through Ampere’s law taking note of the dipole-dipole interactions contributing to the realism of the study, but conditions that could influence heat generation in biological environments. Heat generated by the GMNPs due to external alternating magnetic field (AMF) was predicted using the well-established linear response theory (LRT). Findings of the study revealed that gold coated magnetic nanoparticles vibrated when exposed to external magnetic field. This vibration dissipated heat and increased tumor temperature form 37°C to 42.3°C at the center, hence the 90–99 % tumor damage at the tumor center more than at the outer boundaries. Increased heating time of this nanoparticles increased the fraction of tumor damage at the center. Nanoparticle heating efficiency can be experimentally evaluated considering non-uniform dispersion of GMNPs for further expansion of the study.

González and team [57] studied the potential of hydrogel encapsulated liposome-MZI conjugate for localized drug delivery after breast tumor surgery. A comprehensive mathematical model simulated time dependent liposome internalization and transport in 3D breast tumor. Liposomes embedded in thermosensitive hydrogel that solidifies at body temperature slowly released liposomes overtime, allowing sustained drug delivery directly into the surgical cavity. Functionalization of liposomes with low-density-lipoprotein receptors enhanced uptake and ensured localized targeted delivery to the tumor cells more effectively. A dose- and time- dependent cytotoxicity was observed, with higher concentrations causing greater cell. These results were validated through the Lag exponential death model which accurately predicted cell death based on drug concentration and exposure time. Different liposome concentration at the initial phase has no effect on death rate because the exposure time is long enough to induce cell death through apoptosis. Liposomes remained stable for over 80 days and were effective in killing 90 % cancer cells at the lowest concentration of 0.1 %. Therefore, the rate-limiting step is the release of liposomes from the hydrogel not their movement through the breast tissue. This implies more focus should be put into optimizing hydrogel composition to increase the release of the liposomes into the tissue. This study specified that each tumor geometry and location have a specific hydrogel morphology, size, and liposome concentration that can exert maximum therapeutic efficacy. This aspect could be optimized further in clinical trials of the drug.

Clearly, nanoparticles are the greatest exploration in the cancer fluid dynamics on drug delivery. Studies that employ 2D simulations oversimplify the TME and the fluid flow in the human body hence the slow translation of dancer nano drugs to clinical application. Additionally physicochemical properties of NPs are omitted, which leads to little progress in drug development. An expansion of the models should be applied to different tumors and consider the contributing properties of NPs. Experimental validation can also be a standard incorporated into these studies for validation purposes. Lack of accurate models of complex delivery processes and absence of model parameters for tissue properties are the major barriers to drug delivery modeling. These limitations will be even more pronounced for those drugs that undergo complex bioreactions *in vivo*. Therefore, mathematical modeling can only provide qualitative analysis. For improvement, specific models can be developed to describe a certain drug delivery process based on the findings from biochemical studies (Table 3, Table 4, Table 5).

**Table 3:** Summarized nanoparticle physicochemical behavior in targeting cancer drugs to the tumor.

**Drug Delivery Factor**	**NPs Parameters**	**Results**	**References**
Release rate	Concentration	• Gravity increases liposome release from the hydrogel. • Differences in concentration do not have a significant therapeutic impact. • Low liposome concentrations are sufficient to reduce cell viability to 10 % over a month	[57]
Dual function	Concentration and Magnetisms	• Magnetic field regulates shear stress, which affects how nanoparticles interact with vessel walls and tumor tissues. • Guidance of the NPs with magnetic fields increases concentration near tumor sites. • NP-H_2_O_2_ reaction reduced NP concentration near tumor tissues but enhances drug activation.	[52]
Clustering	Total mass/concentration	• Clustering in the tumor leads to slightly higher temperature than a homogenous distribution	[50]
Thermal Dissipation	Saturation magnetization, damping coefficient anisotropy	• At room temperature the heating efficiency of the nanoparticles is unaffected	[55]
Thermal Dissipation	Concentration	• Higher nanoparticle concentration leads to more effective heating in the tumor.	[56]
Margination and adhesion Efficiency	Size and shape	• Large particles have a larger probability of being marginated than small particles. • Spherical particles marginate better than ellipsoidal particles. • Ellipsoidal particles have a superior adhesion efficiency as compared to spherical particles.	[28]
Convection enhanced	Drug type	• Drugs with lower blood drainage showed better retention in tumor tissue. • All drugs responded similarly to changes in tissue permeability. • CED is highly localized resulting in lower drug concentration in the normal brain tissue than in tumor tissue	[58]
Cold responsiveness	Concentration	• Combined treatment, cryosurgery and chemotherapy enhances therapeutic efficacy as compared to cryosurgery alone. This treatment increased tumor killing volume while it reduced normal tissue killing volume indicating a more targeted approach	[59]
Tumor Penetration	Type	• AuNPs penetrate the tumor tissues through the extracellular pathway. • Approximately 60 % of intratumoral nanoparticles attach to cells while 40 % attach to the extracellular matrix. • Higher nanoparticle concentrations lead to larger aggregates and reduced penetration efficiency due to increased collision	[60]
Convection enhanced delivery	Free drug vs NP	• Liposomes encapsulated doxorubicin accumulation and penetration into the brain tumor is enhanced as compared to direct infusion. • Liposome encapsulated Doxorubicin has less elimination rate, increased vascular permeability, has enhanced average concentration, penetration, and distribution in the distal tumor regions.	[62]
NP capture in the tumor	Magnetic field	• Magnetic particles effectively directed to tumor spheroids using an external magnetic field	[66]
TransVascular Transport	Size and Polydispersity	• Larger nanoparticles above 80 nm, showed higher specificity for tumor tissues due to enhanced EPR. • Increasing hematocrit percentage increase nanoparticle dispersity and their velocity in the vessels • Delivery is reduced in polydisperse samples with 100 nm and 160 nm populations, conversely, in polydisperse samples around 70 nm, there is a slight increase in delivery to normal tissue. • 100 nm NPs have a greater specificity score compared to 160 nm, implying these NPs may be more suitable for optimal delivery to provide only marginally reduced specificity but greater delivery efficiency.	[68]
Blood brain barrier Permeability	Size Magnetic field	• Magnetic field enhanced the permeability of BBB for magnetic nanoparticles. • Larger magnetic nanoparticles (around 100 nm) show up to a 45 % increase in permeability under magnetic field. • Smaller magnetic nanoparticles (around 10 nm) are less influenced by magnetic field and show relatively unchanged permeability	[69]
Retention time	Type	• Silver nanoparticles and copper oxide nanoparticles showed different retention times in tumor and normal capillaries. • Both silver and copper oxide nanoparticles demonstrated better cytotoxic activity against tumor cells compared to normal cells • Nanoparticles reached tumor cells faster than normal cells.	[74]
Drug distribution	Size	• Smaller cytotoxic nanoparticles cause tumor regression at both low and tumor porosity while larger nanoparticles require high porosity to cause tumor regression	[13]

**Table 4:** A summary of the influence of tumor properties on targeted drug delivery with nanoparticles.

Tumor Properties	Results	Reference
Temperature	• Temperature increase is highly dependent on nanoparticle concentration. • High temperatures damage healthy tissue due to high thermal conductivity • Small capillaries in the microvascular network have a minimal cooling effect on blood perfusion	[50]
Blood Perfusion	• Tumors exhibit limited blood perfusion enhancement under hyperthermic conditions compared to healthy tissues. • The computational framework validates the findings by achieving therapeutic tumor temperatures with significant overheating of healthy tissues.	[55]
Thermal conductivity and Blood perfusion	• Tumor depth and diameter affect heat distribution during treatment. • Abnormal blood flow in tumor affects heat transfer. • Tumors have different thermal conductivity as well as density and specific heat as compared to normal tissues. • Higher metabolic activity of tumor cells generates more heat.	[56]
Vessel diameter and Pore size	• Hepatic tumors showed more efficient nanoparticle transport than pancreatic tumors • Larger vessel diameters and cell pore size facilitate higher nanoparticle velocity and lower pressure gradients. • Increase in vessel diameter increases blood flow velocity and decreases pressure.	[67]
Lymphatic drainage and Cellular uptake	• Liposomes are taken up by cells via LDL receptor mediated endocytosis. • Lymphatic drainage is relevant only after 80 days, initially cellular uptake dominates	[57]
Heterogenous microvascular network and necrotic core	• Drug concentration in necrotic areas is very low with only a small amount of the drug penetrating these regions by diffusion. • Approximately 69.03 % of cancerous cells are killed after treatment with Doxorubicin	[61]
Tissue hydraulic permeability	• High blood pressure influences fluid gain and enhances drug deliver. • Less permeable tumors retained a higher spatial averaged drug concentration but the accumulated in limited regions. • There is more uniform drug distribution and deeper drug penetration with a higher tissue permeability. • Blood flow in ventricles has an effect in tissue permeability than cerebrospinal fluid.	[58]
Heterogenous brain tumor environment	• Antiangiogenic drugs effectively reduce tumor microvascular density which prevents drug dilution and loss by blood drainage. • Combination therapy improves delivery outcomes of cytotoxic drugs	[63]
Tumor Microvasculature	• Heterogenous microvasculature influences drug distribution within the tumor. • Areas with lower microvasculature density receive more drug while regions with higher density receive less	[62]
Interstitial fluid pressure and Blood flow velocity	• Transport efficiency of nanoparticles decreases with increasing interstitial fluid pressure. • Nanoparticles cannot cross the blood vessel wall when the IFP approaches the blood vessel wall pressure. • Transport efficiency of nanoparticles first increases with blood flow velocity and then decreases	[94]
Tumor progression and capillaries	• Tumor progression modulates drug vector distribution in tumor-associated capillaries. • Both particle sizes shared 80–90 % common flow areas of 2–9 % for 0,5 µm particles and 2 µm particles • Dysfunctional capillaries with no flow, resulting from tumor progression, limit access to all particles, making diffusion the prevailing transport mechanism	[44]
Heterogenous tumor environment	• Nanoparticles require a high porosity extracellular matrix to cause tumor regression. • Post-injection Trans Vascular fluid velocity depends on matrix porosity and the size of the drug used	[13]
Nanodrug extravasation in the tumor vasculature	• Increasing IFP inversely impacts nanoparticle transport efficiency. • Nanoparticle transport efficiency first increases with blood flow velocity and then decreases	[26]
Heterogenous microvasculature distribution	• Extra- and intra-cellular drug concentrations in the liver tumor are non-uniform due to heterogeneous distribution of tumor vasculature. • Drugs accumulate faster in well-vascularized regions, where they are also cleared out more quickly, resulting in less effective tumor cell killing in these regions	Zhan Wen Bo 2014
Tumor Geometry and microvascular network	• Spatial-temporal drug distribution shows the complexity of the capillary network is the main factor for non-uniform drug distribution in the tumor. • Drug distribution in different areas depends on the structure of the capillary network and presence of the parent vessels, so controlling these parameters can improve drug delivery. Examining the different pressures of the parent vessels, the difference between the highest and lowest FKCs is about 7.37 %. • Eradicating circular tumors is easier than eradicating elliptical tumors of equal area. • Reducing the size of larger tumors is easier than shrinking smaller ones	[65]
Blood flow	• RBCs enhance distribution of NPs within capillaries improving their delivery to the vessel walls. • Brownian motion dominating vessel wall interface allows nanoparticles to diffuse closer to the vessel wall and bind more effective. • Different hematocrit levels affect nanoparticle dispersion and velocity.	[68]
Nanoparticle that crosses the Blood brain Barrier	• Increased blood flow improves the permeability of BBB for MNPs, with up to a 15 % improvement observed. • In a low area ratio, the percentage of the crossing MNPs presents a difference about 30 % when magnetic field is applied enhancing BBB permeability	[69]
Fluid flow and mass transport	• The thickness of the astrocyte and endothelial cell layers affects the concentration of aptamers delivered to the brain. • Drug delivery efficacy was found to be low, primarily due to mass transfer resistance across the BBB layer. • Transport mechanism changes from convective mass transport in the capillary layer to mixed convection and diffusion in the porous layers, and finally to diffusion in the brain parenchyma	[70]
Heat mass transport in the interstitial fluid	• Heat transfer by radiation affects nanoparticle delivery and heat flux. • Enlarging vessel wall pores enhances drug delivery to deep tissues. • Increased blood perfusion decreases tumor temperature, reducing hyperthermia and photothermal therapy efficacy	[72]
Blood flow in a tumor vessel	• Larger vessel wall pores enhance nanoparticle delivery to the tumor but also increase heat transfer. • Injecting nanoparticles into the blood improves drug delivery to tumors, but also enhances heat transfer, potentially reduces hyperthermia treatment efficacy. • Increasing nanoparticle extravasation coefficient enhances the blood temperature while concentration of the nanoparticles is diminishing. Hence increasing nanoparticle delivery to the tumor but removing more heat from the tumor	[71]
Tumor porosity and vessel diameter in mild hyperthermia treatment	• There was a 10 % improvement in the fraction of killed cells with liposomal drug delivery compared to conventional chemotherapy. • Applying microwave with pulsating power helps avoid tissue necrosis while achieving the necessary temperature for nanodrugs	[73]
Tumor microenvironment and blood flow	• Larger vessel diameter and high blood velocity enabled silver and copper oxide nanoparticles to quickly distribute in tumor	[74]
Tumor Heterogeneity and Drug distribution	• More nanoparticles were trapped in the outer well-perfused tumor region compared to the inner semi-necrotic domain due to higher vasculature density and number of traps in the outer region	[75]
Blood and Interstitial fluid flow	• The velocity and concentration of the nanofluid are higher in the blood vessel and decrease as it moves through epithelial spacing to the tumor • Nanofluid EPR is enhanced as it flows from the blood vessels to the tumor interstitial.	Suleman and Riaz 2020
Tumor necrotic region	• Tumors with larger necrotic regions that are over 20 % of tumor radius, show lower heat generation making treatment to be challenging. • Only 15.5 % of MNP concentration distributed to necrosis with 50 % of tumor radius, resulting in insufficient temperature for tumor ablation. • Larger necrosis areas impede drug penetration, leading to lower heat generation by MNPs.	[77]

**Table 5:** CFD-ML hybrid studies that predicted nanodrug delivery to cancer tumors.

Aim	Modeling	Key findings	Limitations	References
To model blood flow containing magnetic nanoparticles used for cancer therapy.	• 2D blood vessel and magnet model • Three Ensemble models (Random Forest, ExtraTree, AdaBoost Decision Tree) trained and optimized with Whale Optimization Algorithm for hyperparameter tuning.	• ExtraTree is the best performer followed by Random Forest followed by AdaBoost and DT • Magnetic fields can significantly alter flow patterns, enabling precise control over nanoparticle movement	• ML models not applied to other vessel shapes, flow conditions, or magnetic configurations. • Lack of experimental or clinical data to validate the predictions. • 2D model does not capture complexity of blood vessels in TME.	[51]
To analyze and predict velocity profile of ferrofluid in blood vessel	• 2D simulation of fluid flow and magnetic field • Develop ML Regression models (Kernel Ridge, Polynomial and Gaussian Process)	• Gaussian Process Regression model gave the highest performance to predict velocity profiles (R^2^= score 0.99603)	• The predictive model only considers three variables: spatial coordinates while other influential factors like viscosity variations, particle interactions or biological responses are not considered.	[46]
To enhance cancer drug delivery by integrating machine learning (ML) with fluid dynamics Modeling in pulsatile blood flow, using nanoparticles as drug carriers.	• ML adopted the Levenberg-Marquardt Neural Network to model and optimize drug delivery. • 2D CFD simulated how pulsatile blood flow and magnetic field influence drug delivery in a porous media.	• ML models accurately predict flow behavior and enhance drug delivery efficiency. • Magnetic fields and porous media significantly influence nanoparticle transport. • Higher Schmidt and chemical reaction parameters improve targeting but reduce nanoparticle concentration. • ANN predictions closely match numerical results, validating the hybrid approach.	• 2D Modeling of blood as a Newtonian fluid overlook its non-Newtonian nature and 3D flow in large arteries. • Assuming nanoparticles are passive carriers omits the active interactions • Assuming uniform magnetic field does not capture real non-uniform field effects	[52]
To analyze the impact of heat absorption and generation on pulsatile flow composed of nanoparticles and impurities such as H_2_O_2_.	• A 2D mathematical model simulated blood flow as pulsatile nanofluid in a porous channel under a magnetic field. • Neural Network ML model trained on three algorithms (Levenberg-Marquardt, Bayesian Regularization and Scaled Conjugate Gradient) using simulation data to predict flow and transport behavior. • Results are benchmarked against existing literature to validate the numerical scheme	• Magnetic field intensity impacts flow velocity and shear stress suggesting potential application in controlling blood flow in arteries. • Reynolds number and Darcy parameter affect flow velocity. • Chemical reaction and Schmidt number causes a remarkable increase in concentration and velocity magnitudes	• ML algorithms successfully predicted and controlled nanoparticle trajectories in the cardiovascular system hence optimized nanoparticle accumulation in cancerous tumours • Magnetic guidance allowed for better control over nanoparticle movement reducing off target effects.	[53]
To model and predict velocity of blood containing magnetic nanocarriers under the influence of an external magnetic field.	• CFD modeled 2D blood flow and magnetic field interactions using Navier-Stokes and Maxwell’s equation. • DT, KNN and GB, KNN models adopted for predictions and optimized by the Rain Optimization Algorithm	• KNN model achieved the highest predictive accuracy. • Velocity distribution matched CFD results confirming model validity	• Simplified vessel geometry and steady flow assumptions • Biological factors are ignored as inputs.	[54]

### 3.2 Tumor characteristics investigated with CFD simulations for targeted nanoparticle delivery

Abnormal features of the tumor and the complex tumor microenvironment affect transport of nanoparticles into the tumor. Heterogenous tumor microenvironment encompasses abnormal vascular shapes, variable tumor shapes and high IFP gradient are extensively studied in targeted nanodrug [61], [85]. Computational fluid dynamics enables simulation of nanodrug transport and distribution robust tumor models [65].

### 3.3 Effects of tumor size, shape, and necrotic core in nanoparticle delivery

Tumour size and necrotic core have a significant impact on effective drug delivery. Drug penetration is dependent on blood supply and interstitial pressure, these factors are directly dependent on tumour diameter for efficient drug delivery. Tumour shape is also a factor to be considered in developing efficient cancer nanodrugs. Fraction of killed cells (FKC) depends on tumour size, nanoparticle binding affinity, drug degradation and concentration, as well as the tumour necrotic core. Through a multiscale mathematical model incorporated with image-based computational simulation it was revealed that a smaller necrotic core has a high drug concentration and FKC. However, drug dosage and binding affinity have a threshold where there FKC is high, low, or non-existent depending on certain increment or decrement values [61]. The binding site barrier is a known limitation in drug delivery acknowledged by the authors but not incorporated as a therapeutic efficacy metric of MNPs. A static tumor geometry and dependence on single from literature does not represent the diversity of tumor morphologies across patients. (Kashkooli et al., 2020). A noticeable drug penetration is observed in tumors with 10 % necrosis while larger necrotic areas result in near-zero MNPs penetration. The highest MNPs concentration in the necrosis area is about 45 min after injection with 60 % of accumulated MNPs [77]. Authors here have uniquely combined MNPs distribution with temperature field analysis in necrotic core, this highlights the importance of necrosis geometry in treatment planning. However, the necrotic region of the tumor assumed to be static and spherical which does not reflect real tumor morphology. In general, [86], [87] revealed that concentrations usually remain high in the peripheral blood circulation surrounding the tumor but almost no drug is present in 90 % of the tumor core. This subsequently leads to unsolicited outcomes like tumor regrowth since most of the tumor cells remain unaffected by the drug [39].

The geometry of the tumor influences IFP and IFV, together with drug distribution due to complexity of the capillary network. A multiscale model simulated drug delivery to solid vascularized tumors [61]. In comparing circular and elliptical tumor shapes with the same area it is mentioned that treating circular tumor (FKC is 37.87 %) is better than elliptical tumor (FKC is 34.71 %). This is evident in greater side effects in the circular tumor than those in the elliptical one, and these results are different for tumors with different microvascular network. Circular shapes may allow for more uniform drug penetration while elliptical shapes created barriers to effective diffusion and convection od the nanodrugs hence the results. The study mentions side effects but does not elaborate on their nature or clinical significance. A 1.88 % increase of drug might be negligible or critical depending on the context of therapy [65]. In future the study can evaluate the effect irregular tumor shapes (lobulated or spiculated) have on drug delivery compared to elliptical and circular tumors.

### 3.4 Influence of Tumor vasculature in nanoparticle delivery

Microvasculature plays a crucial role in facilitating drug delivery to tumors [88]. Microvasculature organization depends on tumor type, therefore different tumors have different vascular sensitivities. Tumor blood vessels are leaky and poorly disorganized. This is the reason driving incorporation of microvasculature variations in blood flow simulations, either as single vessel or capillary network models [43]. Tumor vasculature is heterogeneously distributed, elongated, tortuous [89].Tumor tortuosity and hyperpermeable vessel raises interstitial pressure within the tumors [90], [91]. Cancer cells in tumor tissues need access to blood vessels to regrow and metastasis. A compromised blood supply and interstitial hypertension renders it a challenge to deliver therapeutics to solid tumors [45]. Therapies targeting tumor vessels are a point of focus to destroy tumor tissues [45].These antiangiogenic drugs are co-delivered with cytotoxic drugs to normalize the tumor microvasculature [92]. A study by Mulpuru [74] compared the behavior of silver and copper oxide NPs in both tumorous and abnormal tissue blood vessels. The nanoparticles had shorter retention in tumor angiogenic vessels as compared to normal vessel. This means nanoparticles reach tumor cells faster which supports targeted delivery. Results also revealed that showed that larger vessel diameter and high blood velocity enabled silver and copper oxide nanoparticles to quickly distribute in tumor due to high vessel permeability.

Sparse microvasculature helps reduce blood drainage causing hypoxia and impaired drug delivery. Cancer research intensifies exploration of antiangiogenic drugs that enhances formation and growth of new blood vessels; reduces tumor microvascular density and potentially improves drug delivery to tumors. A study by Bhandari and team [63] investigated the combined CED of antiangiogenic drugs and liposomal drugs in a heterogenous brain tumor environment using a DCE-MRI-based transport model imported into mathematical simulations. The model reported that coupling antiangiogenic BEV with cytotoxic TMZ offered effective TMZ in the form of high concentration in a dense microvasculature. BEV reduced microvasculature surface area and microvascular density. This subsequently increases extracellular space for drug accumulation and enhances TMZ penetration into the tumor. The effect of microvascular density in transporting antiangiogenic liposome-dox conjugates investigated by Zhan [62] showed that the concentration of liposomal doxorubicin decreases in concentration at lower infusion rates in tumors that have less microvasculature density. This is because reduced exchange area decreases blood elimination by blood drainage and the reduced fluid loss inhibits doxorubicin dilution in the extracellular space. A realistic 2D mathematical model developed by Zhan and colleagues [88] showed that heterogenous blood vessel distribution has a strong influence on doxorubicin concentration in blood plasma. Following drug administration, encapsulated doxorubicin concentration in the tumor increases rapidly during the initial period and reaches equilibrium with blood plasma depending on vascular density. The simulation by Bhandari and team [63] provides a more realistic modeling and clinically relevant model as compared to the other two authors. In their model they used real patient imaging data from DCE-MRI of two patients. Though the model simplified antiangiogenic effects, detailed spatial variation in microvasculature, porosity and cell density is incorporated in the model. Despite the use of MR images to create 2D and 3D models by [88] and [62] respectively, lack of experimental validation limits the credibility of the models. In future tissue deformation and liposome uptake modeling is recommended to study the intracellular delivery dynamics as well as the effects of backflow on drug delivery.

To assert the theory of vascular specificity in tumors, it was reported by [67] that nanoparticle flow in blood has a positive correlation with vessel diameter but a negative correlation with pressure distribution for both pancreatic and hepatic tumors. Nanoparticles travel faster in hepatic tumors compared to pancreatic tumors due to larger vessel diameters in hepatic tumors. This suggests that nanodrugs may reach malignant tumors more efficiently than benign ones. Blood components such as red blood cells and plasma also influence nanodrug transport and distribution in the tumor tissue. In a study by [44], it was shown that tumor microstructure affects nanoparticle distribution in the tumor area. This study revealed that in the absence of red blood cells 0,5 µm particles had a significantly larger specific accessible area to the tumor as compared to the 2 µm particles. The presence of red blood cells increased accessible circulation area of the smaller nanoparticles as compared to larger particles, especially in more ordered flows. The effect of red blood cell on nanoparticle delivery to tumors was also investigated by [68]. The model in this study assumed laminar flow of red blood cells in their native biconcave disk shape. This deformation into parachute conformation affects distribution and fluid dynamics of particles in the fluid phase of blood. In regions with red blood cells there was a significant increase of flow velocity that enabled nanoparticle displacement by Brownian forces. At 0 % hematocrit, there is an even dispersion of nanoparticles but increasing hematocrit increased nanoparticle dispersion indicating that red blood cells enhance nanoparticle dispersion in tumorous vessels. A study by Kiseliovas and team [44]provided a multimodal methodology that integrated microfluidics, intravital imaging and CFD to highlight the importance of vascular architecture in NP delivery to tumors. While this study produced quantitative insights into drug delivery, NP-tumor interaction is not modeled. Fullstone and colleagues [68] used an Agent-based modeling framework to simulate NP behavior under blood flow in capillaries. ABM and inclusion of Brownian motion is an innovative approach to simulate complex flow dynamics and NP-capillary wall interaction. This because it captured microscopic flow patterns capturing emergent behavior from individual particles which is more realistic than macroscopic models. In another study, [67]simulated transvascular NP transport in liver and pancreatic tumors to examine how variations in vessel diameter and endothelial pore size affect velocity and pressure gradients. The study only focused on physical transport and lacked biochemical and cellular interaction consideration. Collectively, these studies underscore the complexity of NP transport in TME and emphasizes the need for integrative modeling approaches. While physical parameters such as flow dynamics and vascular architecture are critical, future models should account for biochemical interactions such as receptor binding, immune response, dynamic tumor growth to study tumor-host interaction in biologically complex and variable realistic tissue architectures.

High plasma concentration, high microvascular density and drug extravasation enhances drug concentration in the tumor [65]. A multi-scale mathematical simulation of drug delivery to solid tumors considered microscopic and macroscopic scale delivery on a vascularized realistic tumor geometric model. The study revealed that at high plasma concentration and application of pressure on the parent vessels, nanoparticles increasingly penetrate the tumor extracellular space leading to an increased FKCs. In this study, free drug gradually turned into bound drug that increasingly penetrated tumor intracellular space leading to a high fraction of killed cells [65]. While the authors capture the complexity of drug delivery mechanisms through cellular uptake and interstitial transport, the lymphatic system is assumed to be ineffective in tumors and relies on literature without patient specific calibration of some parameters (such as permeability and osmotic pressure). A 2D mathematical model provided a simulation of blood and nanoparticle transport in a single vessel surrounded by a tumor. In this model, blood is described by the Navier-Stokes equation coupled to advection-diffusion equation, and the energy equation for fluid and nanoparticle transport was developed to evaluate leakage of solute flux into the targeted tumor or healthy. This model depicted that Increasing nanoparticle concentration in a tumor vessel increases blood viscosity, in turn mixed convection, temperature distribution and flow is reduced while nanoparticles accumulate in the tumor. Nanoparticle in blood circulation accumulate in the tumor where blood vessel wall is porous hence a decrease in nanoparticle concentration along the vessel [71]. This model can be extended to include dynamic NP behavior and non-uniform vessel geometries to increase therapeutic credibility.

### 3.5 Effect of tumor biophysical properties and external factors in targeting nanoparticles to the tumor

Tumors have high IFP [93] as compared to normal tissues and that hinders transvascular transport of nanoparticles which is important is important for determining drug transport in tumor. Interstitial fluid pressure greatly influences drug perfusion which in turn affects the extent to which the drug penetrates the tumor [39]. Therefore, studying how IFP affects nanodrug accumulation is paramount in developing effective cancer nano therapies. Hydrostatic and osmotic pressure are key components of interstitial fluid homeostasis in the tumor [90]. A study by Gao and colleagues [94] showed that increase in IFP decreases nanoparticle transport efficiency and reduces number of nanoparticles that crosses the blood vessel into the interstitial domain.

Blood flow velocity and membrane porosity have a significant effect on drug delivery process to tumors. Li and fellow researchers [26]selected a Eulerian-Eulerian two-phase mixture model to investigate transport of drugs numerically and experimentally. The researchers mentioned that drug concentration distribution in the tumor increases with time when the flow rate is kept constant. This might be attributed to a positive correlation that exist between flow velocity and drug concentration in the tumor. This revelation was also supported by [70], where increasing inlet blood velocity promotes increased aptamer concentration and distribution in the blood brain barrier. This is because increasing velocity enhances convective mass transfer. In their study, [26] offered clear insights into how physical factors influence drug delivery through the systematic study using a two-phase mixture model in a microfluidic set up. However, the study should extend the model to 3D simulations and incorporate TME components for more realistic flow dynamics. Sarafraz and team [70] included all major cellular layers and the neurovascular unit to produce realistic BBB presentation. Additionally, the model used a simplifies axis-symmetric 2D geometry that does not fully capture the 3D complexity of the BBB, therefore 3D BBB models with realistic features are recommended for future research.

Yang and Zhan [58] examined the effects of tumor tissue hydraulic permeability on nanodrug-mediated CED. A 3D realistic tumor model from a patient MR image modeled interstitial fluid flow and drug parameters (transport, release, binding, degradation, and elimination). Their findings report that higher tissue permeability leads to uniform drug distribution and drug penetration into deep lying tissues. This covers a larger effective tumor volume for cell killing. Additionally high blood pressure strongly influences IF pressure and flow leading to improved drug delivery outcome in this scenario. Cross-comparison shows the delivery outcomes are more sensitive to changes in tissue hydraulic permeability and blood pressure than fluid flow from the brain ventricle. Spatial average drug concentration is achieved in less permeable tumor, but the drugs accumulate in limited regions leading to smaller area of distribution. In normal tissues, drug concentration depends on hydraulic permeability of tumor tissue because fewer drugs can escape the tumor of the tumor when flow resistance is high. This demonstrates that CED delivery outcomes are highly localized. Despite the novelty of combining CED and tissue hydraulic permeability, lack of experimental validation, over simplified assumptions, missing or omitted details on biological processes and nano drug properties, lack of correlation with actual patient outcomes limits the model’s potential application for clinical translation. Microscale research and advanced medical image should be adopted to address the complex brain interstitial fluid flow mechanisms and how that affects drug-tissue interaction.

Drug delivery systems involve different transport mechanisms, such as convection and diffusion mass transport. Convection-enhanced delivery improves treatment by facilitating a favorable hydraulic environment for drug penetration and accumulation. Diffusion allows drugs to move through interstitial space to reach cancer cells [62]. Increasing mass flux across the tissue increases number of nanoparticles leaving the tissue and reduces nanoparticle concentration in the tissue. In this case, nanoparticles accumulate in the tumor and surrounding tissues [72]. A Multiphysics model applied to 3D realistic brain tumor extracted from MRI demonstrated that CED of liposomal doxorubicin depends on improved interstitial fluid flow. This achieves high drug concentration and large effective delivery volumes in tumors with low microvasculature (Zhan & Wang, 2018) [62]. A change in mass transfer from convection to diffusion leads to decreased aptamer concentration in the basement membrane of the brain. This change in transport mechanism is a result of increased endothelial cells, pericyte and astrocyte thickness decreased aptamer concentration in the basement membrane of the brain [70]. The studies provide unique insights into drug delivery challenges and strategies, however coupling the models with systemic pharmacokinetics and pharmacodynamics with patient specific vascular maps to simulate heterogeneity in the blood vessels would increase the potential of advancing the studies to translational and clinical research.

Temperature control in the tumor is also an important point to factor in targeted nanodrug delivery studies. Varying blood flow and tumor heterogeneity are obstacles in maintaining uniform temperature in the tumor. Different studies demonstrated that temperature distribution influences the amount of drug released, concentration and tumor penetration capabilities. For example, Adabbo and team [73] developed a computational 3D analysis of liposomal drug delivery to investigate the effectiveness of thermosensitive liposomes therapy. It was revealed high binding affinity of doxorubicin increased its concentration which enhanced treatment efficacy. The increase in concentration is because hyperthermia induces liposomes to open releasing doxorubicin. This leads to increased FKCs indicating that coupled treatment is more efficient than conventional treatment. Ismaeel and colleagues [72] mentioned that increasing heat flux towards the vessel decreases the nanoparticle concentration in the tissue, while heat flux increases across the tumor edge increase nanofluid temperature. It is therefore established that either thermal or photothermal therapy could be more effective in cancer treatment. While Mansour and team [71] incorporated thermal effects such as thermophoresis and radiation which a relevant in hyperthermia-assisted drug delivery, the vascular geometry is highly idealized as a cylinder, this does not reflect the irregular and tortuous nature of tumor vasculature. The combined microwave hyperthermia in TSL-based drug delivery by [73] is a promising alternative to conventional chemotherapy even though blood flow dynamics in the vascular system are oversimplified.

Nanofluid temperature drops exponentially in regions adjacent to the vessel wall, this reduces fluid extravasation to the tissue. Tissue temperature is maintained, subsequently enhancing tumor thermal therapy. Increment of IF extravasation velocity at the vessel wall improves nanofluid transport in the extracellular matrix and increases nanoparticle concentration in deep tumor tissue [76]. A study by [71] showed that as temperature distributions in the circulatory system diminished nanoparticle concentration is enhanced and blood becomes viscous, but increasing thermal radiation enhances heat flux and temperature differences within the flow domain and thus enhancing convective transport of nanoparticles. The authors model and simulate heat and nanoparticle transport through Brownian Motion, thermophoresis, thermal radiation, and NP extravasation. However, the assumption of a steady state heat transfer does not reflect the dynamic nature of tumor tissues especially during hyperthermia treatment or drug delivery. Therefore, transient analysis to capture time dependent temperature changes during treatment, and incorporation of perfusion effects using Penne’s Bioheat model together Local Thermal Non-Equilibrium (LNTE).

Model geometries for 2D simulations are always assumed to be spherical, this limits delivery of drugs to tumor sites. In an experimental set up of Hyperthermia mediated treatment in the tumor temperature fields cannot be measured, and subsequently the influence of nanoparticle clustering. Therefore, simulations give valuable insights that experimental studies cannot. In conventional models, blood perfusion is treated as a fixed parameter characteristic of specific tissue types, disregarding its physiological response to temperature changes. However, perfusion varies with temperature, particularly in tumor tissues where vascular regulation is impaired. The assumption of a constant perfusion rate leads to an underestimation of the tumor’s temperature rise during hyperthermia. This could result in an inaccurate assessment of treatment efficacy, as the thermal dose delivered to the tumor would appear lower than what occurred. In cancer modeling a porous-media model is often adopted to capture interactions in the TME where the solid phase is considered as the ECM and IF fluid is modelled as the fluid phase in the pore space. The cells (tumor and host) are modelled as highly viscous fluids than solids. The fluid phases fill and flow in to share the pore space of the ECM forming a porous medium. However, the exact geometry of the ECM is very complex.

In targeted drug delivery, magnetic hydrodynamic flow models have shown that magnetic nanoparticles (MNPs) generate heat at a target site when a magnetic field is applied, subsequently enhancing their tumor penetration ability. A study by [77] reported that at the tumor center, high temperatures lead to maximum accumulation of MNP and decrease in temperature causes MNP decay. In another study, Gkountas [69] showed that MNP passing through the blood brain barrier depends on rheological conditions and external magnetic force. A gradient in blood flow pressure forces MNPs towards the endothelium, and increasing magnetic field increases the number of MNPs passing through the BBB. In this study it was revealed that a smaller ratio of endothelial cells to nanoparticle decreases nanoparticle permeability hence a reduction in the number of nanoparticles that pass into the blood brain barrier. Experimental results correlated with simulations where it was established that increasing magnetic field applied increases MNP concentration.

Cryosurgery uses extreme cold temperatures (-20°C) to freeze and destroy deep lying tumors. Cold responsive nanoparticles release high doxorubicin amounts into the tumors where drug concentration increases with increased cryoprobe holding time. This reduces drug elimination by blood drainage. This increases the effective tumor killing volume by 0.27 % while normal tissue kill volume increased by 70.1 % [59]. Integration of cold-responsive NPs with cryosurgery is a promising strategy to overcome limitations of conventional cryotherapy in peripheral tumor regions. The authors present a highly theoretical study without detailed discussion on systemic toxicity or pharmacokinetics and lack of both *in vivo* and *in vitro* validation. This creates a clinical translation gap. Model validation through experimental data and incorporation of realistic models that explores complex tumour-drug complexities will bridge this gap.

The cooling effect of blood perfusions is poorly understood, and precise heat control is important to avoid damage to healthy tissues. A study by [50] presented a 2D computational model that simulated nanoparticle accumulation and the cooling effect of blood perfusions in hyperthermia-mediated cancer treatment in whole tumors. This study assumed the tumor as a multiphase porous model at a macroscale that captures the physical properties of the TME, their interactions and the transport processes. Hence the used the thermodynamically constrained averaging theory (TCAT) to derive the macroscale equations from the microscale equations. Nanoparticle transport that accounted for extravasation, interstitial fluid flow and lymphatic drainage, was modeled through the diffusion-advection equations. Heat transfer due to diffusion and convection in the tissue, heat generation by nanoparticles, and the cooling effect of blood perfusion was modeled through the Lumped model using Pennes’ bioheat equation and discrete model. Findings revealed that temperature increase reached in the tumor highly depends on the number of nanoparticles accumulated and the specific absorption rate. Temperature fields showed that clustered nanoparticle accumulation *in vivo* increases thermal conductivity of the tissue also affects the surrounding healthy tissues over 52°C. The study successfully demonstrated the ability to optimize hyperthermia treatment by predicting temperature fields and guiding nanoparticle dosage and distribution of heat control in cancer treatment. However, the heat transfer model overestimated the cooling effect of blood perfusion leading to underestimation of tumor temperature. The lumped heat sink method has many discrepancies such as its spatial averaged formulation for heat transfer, blood perfusion that does not consider flow direction and assumes uniform perfusion. Therefore, considered an approximation equation without physically consistent theoretical basis, this requires coupling it with other governing equations to validate the results.

### 3.6 Exploration of extracellular matrix in tumor-nanoparticle interaction

The extracellular matrix and porosity play an important role in drug distribution and penetration. There is research-based evidence confirming that porosity matrix plays a significant role in determining the extent of drug perfusion, with greater porosity implying faster drug diffusion through the matrix [39], [90]. Cytotoxic 1 nm particles can regress a tumor at both low and high porosities while larger nanoparticles required high porosity to cause tumor regression as reported by [13]. A study by Xie and team [60] revealed that cancer cells can adhere 60 % of AuNPs while the remaining 40 % adheres to the extracellular matrix. This was common for different AuNPs concentrations suggesting that nanoparticle collection proportion is inherently determined by tumor tissue characteristics such as porosity. In this study initial repulsion forces are weak for AuNPs-ECM interaction, but these forces become strong with increasing driving AuNP kinetic motion increasing nanoparticle uptake efficiency. A microvascular hemodynamics governed by Hagen-Poiseuille law was adopted to predict the influence transvascular velocity has on nanodrug distribution. Findings showed that for the earliest administration, drug distribution is independent of drug type (nanoparticles (10 and 50 nm) or cytotoxic drug (1 nm)), but later administration showed that transvascular flow depends on drug size and porosity. Cytotoxic drug distribution increased than nanoparticles at the tumor center where there is low porosity. This drives the porosity dependent penetration of drugs. This model revealed that the cytotoxic drug regressed the tumor at both high and low ECM porosity while nanoparticles require high porosity to cause tumor regression [13]. Li and colleagues [26]mentioned that low membrane porosity results in a linear and slow drug concentration in the tumor, but a decrease reduces time taken to achieve higher drug concentration, further increase does not have an effect. Membrane porosity determines mechanism of nanodrug mass transport in the tumor. A study by Sarafraz and team [70] showed that the porous basement membrane enables a mixture of convection and diffusion mass transport in the brain parenchyma and when blood velocity decreases diffusion becomes dominant transport leading to an increased drug delivery [70].

For all nanodrugs (1, 10 and 50 nm) at high ECM porosity, concentration is higher both inside and outside the tumor while at low porosity drug concentration is lower and uniform throughout the volume. Both porosities produce a uniform distribution to the cytotoxic (1 nm) drug but a highly heterogenous distribution of larger drugs. While low ECM porosity reduces the efficacy of all the drug types, cytotoxic drug regressed the tumor at both porosities. Nanoparticles on the other hand require high porosity to cause tumour regression. The model predicted that NPs benefit from later injection times at high porosity, this is due to the elevated interstitial fluid pressure produced by larger tumors increasing the magnitude of convective flow. At the earliest administration, drug distribution is largely independent of drug type and transvascular flow is dependent on both drug size and porosity. This drives the porosity dependent penetration of the drug. The model framework incorporated biochemistry and angiogenesis into solid and fluid mechanics to offer a complete view of tumor-host interactions. Unlike static models, a dynamic tissue approach simulates evolving TME and vascular network overtime. Immune mediated ECM changes, drug- induced vascular remodeling, cellular heterogeneity or tumor evolution are relevant factors not included in this study which can guide drug response [13].

The contribution of blood perfusion in heat distribution in tumors during hyperthermia-mediated treatment with magnetic nanoparticles was explore by [55]. A bioheat transfer model adopted a 2D model that was solved by Finite Element Method in COMSOL physics. The model incorporated blood perfusion as a heat sink term in the tumor and the perfusion rates depended on temperature and time. While this paper does not simulate fluid flow in the traditional CFD sense, it models perfusion as a dynamic fluid exchange process that significantly affects thermal behavior relevant to cancer heat transfer. The results revealed that tumor tissues exhibit limited blood perfusion enhancement as compared to healthy tissues under hyperthermic conditions. This vascular dynamics in turn lead to localized heat retention in tumors for therapeutic purposes. These simulations achieved high therapeutic tumor temperatures without overheating the surrounding tissues. This dependence of blood perfusion on temperature and time in this model advances the capturing of dynamic physiological responses which are limited in static perfusion rates. This model demonstrated the efficacy of combining nanoparticle modeling with temperature dependent perfusion to optimize magnetic nanoparticle-based hyperthermia protocols. The study revealed that underestimating the temperature increase could lead to suboptimal treatment protocols, potentially reducing therapeutic effectiveness. Conversely, an overestimation of heat dissipation in healthy tissues could cause unnecessary concerns regarding thermal side effects. The findings highlight that incorporating a variable perfusion model is essential for improving the accuracy of magnetic hyperthermia (MNH) simulations, ensuring a more reliable prediction of the thermal response, and enhancing the precision of clinical hyperthermia applications. Furthermore, the inclusion of parametric analysis strengthens the study by providing a broader perspective on treatment effectiveness under different physiological conditions. From the bioheat transfer simulations, it is evident that the MNH treatment achieves therapeutic temperatures (41–45 °C) in the tumor without overheating the surrounding healthy tissue. This selective heating highlights the potential of MNH in minimizing adverse side effects, which is a persistent challenge in other cancer treatment modalities like chemotherapy and external beam radiotherapy.

Gold-coated magnetic NPs were demonstrated to be highly effective in damaging cancer cells in (Gas et al., 2025). A 3D tumor model showcased how gold-coated nanoparticles are used to elevate temperatures in tumors leading to effective tumor killing. A 3D artificial skin cancer model was created using finite element in COMSOL Multiphysics to simulate magnetic field distribution, heat transfer and predict tumor damage. The magnetic field was modeled using the Ampere’s law, heat generation mechanism was modelled by Rosenzweig’s model and Penne’s bioheat transfer model while the tumor damage was predicted through the Arrhenius Kinetic model. Their findings revealed metabolic heat generation and the GMNP vibration from being exposed to a magnetic field increases body temperature from 37 to 42.3°C at the tumor center. Continued increase heating time increased tumor (90–99 %). Blood perfusion rate was found to inversely affect heat distribution. Models successfully predicted tumor killing though further validation with *in vivo* and *in vitro* experiments is crucial to demonstrate the efficacy of this nanoparticles in cancer treatment. This model accounts for the reduction in tumor perfusion at higher temperatures, better reflecting the real physiological response and predicting a more localized and sustained heating effect. The implications of this discrepancy are critical for hyperthermia treatment planning.

A team lead by González [57] developed and applied a comprehensive mathematical model for Multiphysics simulating fluid flow through a porous media to optimize localized drug delivery. This model simulated the MZ1-loaded liposomal release and transport from hydrogels for cavity filling following a patient specific breast tumor surgery. Cell viability governed by through the lag exponential death model and gravity influences based on patient posture were taken integrated into the simulation. It was revealed that lymphatic drainage is only relevant after 80 days while cellular uptake dominates the liposomal uptake. This study supports personalized hydrogel design based on tumor morphology, dose optimization and minimized side effects. Two sink terms; uptake and drainage, that contribute to liposome reduction in the tissue were investigated. Cellular uptake dominated liposome reduction as the reaction predominantly followed the direction of mass flow favoring gravity. Initially, there was a substantial disparity in both uptake and lymphatic drainage. As time progresses, both rates are in the same order of magnitude concluding that lymphatic drainage plays a significant role in the availability of liposomes in the breast tumor. Validating the model with *in vivo* models and retrospective clinical data as well as inclusion liposome degradation and drug release kinetics are opportunities that can be considered in the future to improve the model.

### 3.7 Nanotechnology, machine learning and computational fluid dynamics integrated in cancer biomedicine research

Nanotechnology enables the development of nanoparticles that can deliver and release drug directly to tumors without affecting normal cells. Nanoparticles in cancer treatment offer attractive features sought after for cancer treatment increased bioavailability and less off-target effects. Machine learning has been integrated with computational models to enhance the design and delivery efficiency of nanoparticles for targeted cancer drug therapy. Given the inherent challenges in conventional chemotherapy such as damaging healthy cells and the low tumor delivery efficiency (0.7 % of injected dose accumulates in tumors) of nanocarriers, advanced modeling approaches are critical for optimizing treatment effectiveness [95].

Different studies have integrated ML to predict minimal side effects, NP-tumor interaction, and optimal design to advance the translation of nanodrugs to clinical levels. EVONANO platform designed by [96] used ML models to simulate nanoparticle transport through a virtual tumor to predict nanoparticle distribution. A multicompartmental model that integrated nanotechnology, molecular communication, and internet of Bio-Nano Things (IoBNT) was developed to integrate AI-driven bio-cyber interfaces showed great performance in increasing drug concentration only at the target sites [97]. Molecular dynamics simulates incorporated ML to how NP physicochemical properties influences their interaction with biological systems showed that there is a 40-fold speed improvement and 25 % accuracy increase over the existing methods [98]. ML Kernel Ridge Regression model predicted NP biodistribution in a tumor tissue with high efficiency and accuracy as compared to other models.

Machine learning and computational methods are poised to transform the entire pipeline of NP-loaded drugs development, from synthesis to clinical applications. Machine Learning accelerates the search for optimal synthesis parameters and predicts desired physicochemical properties such as drug loading and optical properties. NP-tumor interaction interactions which critically affect blood circulation and systemic pharmacokinetics can be predicted through ML and tumor targeting can also be leveraged through ML by identification of biomarkers that influence NP accumulation such as blood vessel density and tumor associated macrophage density [99]. Combination of ML with imaging techniques assist with quantifying tumor vascular permeability and predict NP delivery to individual micro-metastases [100], [101]. ML algorithms can also predict cellular internalization patterns based on NP properties and optimize NP designs to selective cytotoxicity towards cancer cells [96]. Through research, it has been established that CFD can simulate the complex interaction between drug delivery systems and tumor microenvironment by optimizing treatment and drug delivery mechanisms. This subsequently provide insights into the behavior of therapeutic agents in the human body. It however has its own challenges of over-simplifying the tumor geometries due to lack of model optimization, and lack of validation with both *in vitro* and *in vivo* studies. Integration with artificial intelligence presents a more technological convergence that may address these challenges and optimize personalized delivery formats [99]. The interdisciplinary synergy allows for modeling phenomena such as drug perfusion, tumor induced vascular re-modeling and interstitial pressure gradients with a level of fidelity previously unattainable. AI algorithms such as ML and DL enhances CFD workflows through intelligent automation and pattern recognition, transforming static simulations into dynamic patient specific tools [53]. As a result, ML and CFD integration not only enhances understanding of tumor mechanics but informs personalized therapeutic interventions, shifting cancer care from protocol-based practice to precision-guided engineering of treatment outcomes. Studies that combined CFD and ML models to simulate and predict tumor and nanodrug interactions are summarized in table 6.

Modeling complex systems like ferrofluid motion in blood vessels is computationally expensive when explored through the mechanistic CFD models. A study by Alqarni and team [51] presented a hybrid modeling framework that trained ML models on velocity data extracted from CFD simulations for fast prediction of velocity fields of blood flow in in a blood vessel under magnetic field. A dataset with more than 17 thousand rows and the velocity distribution versus x and y were used to build ML model that simulated blood flow in a blood vessel under magnetic field. Fluid dynamics and magnetic field were governed by the Navier-Stokes and Maxwell’s equations respectively and model optimization achieved by using Whale Optimization Algorithm. Among the three ML models adopted to validate and extend the CFD, high performance and higher predictive accuracy was realized with ExtraTree (R^2^=0.99151) with low errors as compared to Random Forest and AdaBoost Decision Tree. While the study achieved high accuracy, a 2D model made of blood vessels and an external magnet was adopted for fluid flow computations. A 2D assumption simplifies the feature space and improves model training speed and accuracy. This creates a continuum fluid flow which does not display the realistic blood flow with magnetic nanoparticles under external magnetic field. This model also lacks the interpretability of ML models to clinicians, hence the limited understanding of model decisions in biomedical applications. The overreliance of this study on CFD data for training and testing, does not explore model performance under noisy or sparse data. ML-models were not used to replace CFD but to reduce computational cost by predicting velocity fields in regions where CFD simulations are expensive.

In another study, a CFD-ML model was adopted to predict ferrofluid velocity profiles from a data set comprising of x(m), y(m) as input variables and U(m/s) as an output variable [46]. The model assumed laminar/continuum flow governed by Navier-Stokes equations and magnetic field and density resolved by Maxwells’s and Gauss’s equations. Three models regression models; Gaussian Process Regression, Polynomial and Kernel Ridge Regression models optimized Dragonfly algorithm were compared for their predictive accuracy. Gaussian Process Regression model achieved highest predictive accuracy (R^2^=0.99603). From the two studies it is highlighted that selection of appropriate algorithms is of high importance. A combination of both tree and regression models should be combined to reduce overfitting risk, provide probabilistic outputs with uncertainty, and increase interpretability of the models.

Furthermore, [52] reported that a 2D-CFD-ML model to investigate nanoparticle behavior in pulsatile flow modeled using Levenberg-Marquardt neural network in pulsatile blood flow. Magnetic fields included as an external factor in the model increased flow velocity and shear stress resulting in aligned nanoparticle flow in the blood. The magnetic guidance of nanoparticles ensured targeting and uniform distribution at the tumor site. The neural fitting of the ML-models accurately predicted flow behavior and enhanced drug delivery efficiency of nanoparticles (R^2^=0.9981). Even though ML predictions matched those of the numerical simulation, advancing the model to a 3D model and capturing specific nanodrug designs could improve the predictions.

Alsaab and Althobaiti [54] attempted to improve targeted delivery by modeling behavior of magnetic nanocarriers in blood. Simulations were performed on COMSOL Multiphysics with Blood flow and magnetic were modelled using Navier stokes and Maxwell equations. Velocity data (U) was extracted as a function of spatial coordinates (x) and (y). Three models (Decision Tree, K-Nearest Neighbor (KNN) and gradient Boosting) optimized by the Rain Optimizing Algorithm were compared for their predictive accuracy. KNN gave the best performance (R^2^ = 0.99088 concluding that this model is ideal for capturing local velocity variations due to its instance-based learning. Even though the hybrid model offers high predictive accuracy, reduced computational costs and potential for real time control of magnetic delivery system. Future research should extend the model to 3D pulsatile flow with more biological parameters as well as experimental validation.

A hybrid 2D-ML CFD model developed by Akhtar and colleagues [52] integrated ML techniques to enhance magnetic fields for drug delivery process optimization in the circulatory system. The study explored how Drug parameters (magnetic field intensity, Reynolds number, Darcy model, Prandtl number and chemical reaction) affect momentum, thermal, and concentration profiles of ferrofluid flow. A mathematical 2D model uniquely considered the impact of heat absorption and generation on pulsatile flow composed of NPs and undergoing chemical reactions. Navier-Stokes equation with Forchheimer drag terms, energy equations to account for heat generation and thermophoresis, nanoparticle concentration equation with Brownian Motion and thermophoresis, and chemical reaction equation governed the model. A higher Darcy-Forchheimer parameter increased flow resistance, larger Prandtl number affects the heat profile that impacts the thermal distribution. An increase in Schmidt number increased the mass transfer rate decreases and the fluid capacity to carry species is reduced. Increasing the chemical reaction reduced NPs concentration leading to a localized concentration of nanoparticles than even distribution. Additionally, it was established in this study that magnetic field has a greater effect more on velocity than temperature changes. Even though the model adopted a 2D simulation of vessels and blood flow, adoption of realistic fluid rheology (H_2_O_2_ as a biomarker simulated realistic biochemical interactions) enhanced the precision of the estimates. The predictive power of ML-models demonstrated enhanced magnetic targeting that in turn enhanced the precision and effectiveness of nanoparticle mediated drug delivery. This study established significance and credibility of the findings by comparing them with studies in literature.

### 3.8 Predicting the future of converging nanotechnology with computational fluid dynamics in cancer biomedicine research

Combining nanoparticles, computational fluid dynamics and machine learning in cancer therapy expands and transforms the cancer biomedicine research. Each of these domains are isolated to focus on narrow slices of the research area. Convergence of this fields of research enables a systems-level approach to cancer therapy where physics, biology and data science holistically design smarter, faster, and more effective cancer treatment [99]. Targeted drug delivery effected by nano-vectorized drugs offers precise delivery of drugs to tumor sites by exploiting tumor-specific markers [95], [102], [103]. CFD simulates blood flow and nanoparticle transport to optimize delivery routes and predict accumulation zones while machine learning learns from patient specific data to personalize delivery strategies and predict treatment outcomes [96], [99]. With their integration researchers can model the entirety of patient-tumor-nanoparticle interaction through facile multiscale models. Future of CFD-ML-NPs hybrid models in advancing cancer targeted therapy are discussed below.

Tumor microenvironment complexity can also be dismantled with CFD’s role to model fluid dynamics within tumor tissues to understand nanoparticle tumor interaction while ML models analyze biological data and imaging to classify tumor types and adapt treatment protocols accordingly. ML models can build predictive models from patient specific data to tailor therapies while CFD simulates personalized fluid dynamics based on individual anatomy and pathology to fill the gap. Additionally, ML models trained on CFD simulations and biological outcomes can predict optimal nanoparticle properties for specific tumor types and vascular environments. ML-CFD hybrid models have also the potential to use patient imaging and biomarkers, while nanoparticle formulations tailored to individual tumor microenvironments.

Computational fluid models are often static and computationally expensive. Real time monitoring and prediction of nanoparticle behavior in the human body is possible with ML models processes imaging and sensor data to track nanoparticle movement and predict therapeutic impact. CFDs can provide dynamic models that can be updated with real time for adaptive treatment planning. One-size-fits-all treatments fails to account for individual variability in tumor biology and patient physiology therefore stalling the advance of personalized cancer therapy. Each field generates its own data, often in silo, however in combination CFD outputs, nanoparticle tracking data, and clinical outcomes can be fused into unified datasets, enhancing ML model accuracy and interpretability. Currently, translating experimental discoveries to clinical use is slow. Predictive modeling and simulation reduce the need for exhaustive *in vivo* testing, speeding up regulatory approval and clinical deployment. Future efforts should focus on integration multi-omics profiles, advanced imaging, and patient specific tumor characteristics into multiscale modeling frameworks to better simulated NP behavior and enhance predictive power and personalized medicine. CFD is acknowledged to be complex and computationally expensive and time-consuming for simulating complex systems. Hybridization with machine learning can help overcome computational limitations experiences with CFD. This is because a hybrid model framework combines mechanistic simulation with data driven ML models. The data extracted with CFD simulations is used to train and validate ML models, creating facile models with high efficiency for simulation fluid flow that affects NP delivery in cancer tumors. Future work will include immune cell modeling and nanoparticle design variations to further optimize delivery. Discussions around the ethical and clinical implications of adopting ML models into cancer drug delivery studies should deeply explored and implemented even in preclinical research.

## 4 Discussion

Descriptive statistics in literature review establishes a foundational overview of the dataset of publications collected. These helps to summarize key characteristics that will facilitate interpretation within the context of existing literature. In this study, frequency distributions of tumor types, nanoparticle type and models adopted in the studies is presented. Additionally, bibliometric analysis identified keyword trends and publications production output. This analysis is crucial as it helps researchers to identify central trends and variability within CFD based simulations for nanoparticle delivery to cancer tumors. Incorporating descriptive statistics into this literature review ensures clarity and facilitates comparisons across studies, ultimately strengthening the interpretation of evidence in fluid dynamics influence to targeted nanoparticle delivery to tumors.

In Fig. 3, the low output from 2014 to 2018 and 2023 can be attributed to funding or resource constraints. A shift towards preparing major projects for later years rather than publishing smaller studies can also be a possible contributing factor to the low output. The surge from 2019 to 2022 and 2024–2025 can be due to completion of large-scale projects initiated earlier. Increased collaboration and adoption of advanced tools such as AI and DEM-DLVO to simulate nanoparticle-tumor interactions. Fig. 4 clearly stipulates that Wenbo Zhan has the largest and darkest dot size indicating that they are the most productive and highly cited in 2022. Other authors such as Farshad Kashkooli have smaller dots spread across multiple years indicating consistent but lower outputs. Barbara Wirthl and Benhard A. Schrefler show multiple years of activity but smaller citation impact.

Keyword co-occurrence in Fig. 5, Fig. 6 reveals that the studies are related to medical or pharmaceutical research, with emphasis on drug delivery, cancer treatment, and computational modelling. High-frequency keywords include delivery, nanoparticles, flow, and blood point out themes like drug delivery systems and biomedical research. Lower frequency keyword such as analysis, models, and study suggest methodological aspects are also present but less dominant. The presence of both “tumor” and “tumour” shows variations in spelling. This could be standardized for better analysis. Large size words suggest the main research focus is on drug delivery, tumor modelling, and nanoparticle-based therapies. The medium size words suggest secondary themes like computational modelling and magnetic drug targeting. Therefore, the research is heavily focused on drug delivery systems for tumors, simulation, and modelling techniques as well as nanoparticle applications. Computational and numerical methods are also significant, suggesting a strong link to bioengineering and computational biology. Fig. 7 reveals the thematic focus of the publication in this review. The red cluster has keywords that are likely related to tumor modelling and therapy, the purple cluster keywords are related to machine learning and computational fluid modelling; the blue cluster words are more focused on cancer treatment and hyperthermia. Finally, the green cluster is focused on general computational modelling theme. The dendrogram agrees with the conceptual framework as they show similar thematic groups and keywords. The blue cluster is related to tumor biology and therapy, red cluster is linked to machine learning and drug delivery systems while the green cluster is focused on computational modeling and cancer treatment.

The frequency distribution charts in Fig. 9, Fig. 10, Fig. 11 suggests a trend toward more complex and data-driven modeling approaches such AI and DEM-DLVO models. High magnetic nanoparticle distribution implies that magnetic cancer treatment is extensively studied. Magnetic nanoparticles have enhanced tumor targeting through magnetic guidance in turn overcoming the challenge of poor nanoparticle penetration in dense tumor tissues [104]. Additionally, when exposed to alternating magnetic fields MNPs generate heat that is selective to cancer cell only reducing side effects have unique multifunctional capabilities that enhance targeted precision and therapeutic effectiveness of cancer treatment. Controlled and smart drug release is also one of the attractive features of MNPs because magnetic fields ensure timing and dosage precision only at the tumor site [104]. Metallic nanoparticles (MeNPs) are the least studied because even though these NPs hold great promise in cancer therapy they are yet extensively explored or clinically adopted for several reasons mentioned by [105]. Firstly, metal ions released from MeNPs can be toxic to side effects due to long term accumulation in organs. Secondly MeNPs are unstable and aggregate when in contact with biological fluids. These affects their bioavailability, targeting efficiency and therapeutic performance. Lastly, unlike MNPs or polymeric nanoparticles, MeNPs require extensive surface modification with ligands or polymers to achieve specific tumor targeting [106]. Studies do not specify tumors but rather focus on generic tumors as general solid and vascular tumors are more frequent than other tumors. This generalization limits the expansion of the models into translational research. This is evident in the slow transition of CFD-modelling based research to translational research and clinical trials.

In this review it is evident that physiochemical properties of cancer nanodrugs and tumor microenvironment heterogeneity plays a significant role in determining biophysical tumor-drug interaction for effective and efficient targeted drug delivery. Nanoparticle features such as size, shape and dispersion were investigated against the tumor microenvironment features such as the microvasculature and tumor geometry. Studies in this review have validated that cancer nanodrug delivery depends on their efficient binding, adhesion, and margination to specific targeted sites while distribution, penetration and permeability depends on blood flow properties and blood components (flow rate, red blood cell deformability and hematocrit vessel size and nanoparticle size. Results from studies on different types of tumors and different nanoparticles are consistent, but different. This is owed to different specific parameters in the results such as nanoparticle type, tumor features investigated and the tumor type. Moreover, studies should focus on overcoming TME heterogeneity by using polydisperse in a model instead of one nanoparticle type. A nanoparticle can be investigated for its tumor targeting efficacy by varying size, shape, and surface functionalization to achieve this.

Many of the studies explored magnetic hyperthermia as to investigate potential of magnetic nanoparticle in cancer healing (Fig. 10). A similar trend in all the studies is that magnetic hyperthermia led to tumor regression and increase nanoparticle concentration and distribution in tumor regions. However, there is a limited focus in specification of release kinetics nonthermal effects of as compared to thermal effects because major of these studies are focused on magnetic hyperthermia-mediated cancer treatment [107]. Computational modeling is fundamental to overcome challenges in nanomedicine by particularly determining the influence of physicochemical properties such as size, shape, and surface charges on their biological performance in tumor tissues. This is because these simulations provide atomic-level insights into the dynamics and structural properties of nanodrug delivery to cancerous tumors [108]. Additionally, Current data-driven models are criticized for failing to meet modern design needs, specifically citing the lack of microscopic mechanistic insights. Challenges in developing reliable ML models stem from the lack of standardized experimental data and a deficit of unique nano-identifiers (system-dependent nanodescriptors) needed to describe complex nanoscale structure changes in varying environments [109]. Clinical translation of nanoparticles to drugs is hindered by regulatory and translational barriers created by lack of standardized synthesis protocols, difficulty in scaling production and unclear regulatory pathways for approval [105]l. Therefore, strong collaboration among experts in regulatory and compliance specialists, nanomaterials, oncology clinicians and computer science to integration of robust computational approaches across all stages of research to achieve optimum performance that will assert clinical relevance is recommended.

Numerical simulation is an important approach to investigate drug transport and delivery because of the advantage to decouple and isolate each factor in the series of complex delivery process. Computational simulation of fluid dynamics has enabled developing models that mathematical formulas to assist with visualization of the tumor microenvironment interaction with nanoparticles, and in some studies experimental results are consistent with simulations. Many of the studies have adopted simple 2D simulation as compared to the advanced complex 3D simulations. Even though 2D simulations provide numerical efficiency in a short period of time it limits extensive detailed studies on nanoparticle-tumor interaction in the complex tumor microenvironment. In many of the studies where 2D model is considered over a 3D model, many tumor microenvironment features and tumor progression and geometries are not included. Experimental results coupled with multiphase 3D simulations produced consistent results provided a detailed understanding of the mechanisms of nanoparticle behavior in the tumor that encompasses spatial profiles of velocity, pressure along nanoparticle penetration and distribution within the tumor microcirculation. The influence of the heterogeneous Tumor Microenvironment (TME), specifically the effect of the microvascular network and convective fluid flow on temperature gradients and drug transport during non-thermal and thermal nanoparticle delivery. requires more in-depth study. The reason behind this suggestion is because multiscale and numerical simulations, predictive models have caveats such as oversimplifications of the complex TME and lack of validation experiments [107].

Few studies adopted clinical tumor images coupled with 3D CFD simulation to provide a realistic tumor-host pathophysiology and a model that can estimate optimal biophysical and material conditions for efficient drug delivery. Simulations without realistic images prompts researchers to assume simple parameters/conditions that do not depict the complex *in vivo* properties of tumors while tumor tissues are rather heterogenous than homogenous. Clinical tumor images enable incorporation of the complex and heterogenous patient specific data into CFD simulations that in turn advances the domain of personalized medicine. Inclusion of clinical images of the tumor progression and growth kinetics would enable researchers to develop drug dosages suitable for each stage of cancer therefore developing effective nanodrugs. For further studies, image based CFD simulation should take patient data (pharmacokinetics and dynamics as well as the tumor geometry) into consideration for pharmaceutical scientists to adopt suitable drug design parameters and for oncologists to design specific treatment plans for each patient. Fluid mechanics enabled numerical simulations to configure tumor killing mechanisms, drug delivery dynamics, develop realistic heterogenous tumor models and assessed for drug distribution and dispersion for effective cancer nanotherapy studies. It is evident from the studies in this review that 3D tumor geometry and fluid flow simulation can account for tumor microenvironment at the same time study drug properties such as shape, hardness, and interaction with tumor extracellular matrix thus providing design rules for drug delivery which could complement the existing theories obtained by experimental techniques.

Notably ML incorporation into fluid simulations of drug delivery contributed to reduced computational costs and high prediction of drug delivery to the tumour tissues. The models and algorithms in these studies had optimization constraints that need to be addressed. Numerical and multi-physics models employed in CFD simulation rely on assumptions that oversimplify the highly complex in vivo environment, including factors like tissue heterogeneity, immune responses, and blood flow dynamics, leading to overestimation of therapeutic efficacy compared to real life applications. There also are insufficient and biased datasets that comprise of only a hundred to a thousand datapoints which are inadequate for training complex ML models, particularly deep learning architectures. Standard and biologically relevant descriptors for diverse tumor an NP types are amiss. Furthermore, while tree-based ML models are relatively interpretable, complex neural network models often lack transparency, hindering biomedical trust and clinical translation. Adoption of in silico models such as CFD simulation and ML-based as vital, non-isolated tools are cost effective in driving precise nanoparticle targeted delivery to tumors. This is emphasized in their cost-effectiveness and ability to analyze complex interactions that are often inaccessible via *in vitro* and *in vivo* experiments [107].

Despite the promise of ML and mechanistic computational modeling in cancer nanodrug delivery systems, several critical challenges must be overcome to translate these findings to clinical practice. Firstly, these studies adopted 2D simulations that simplified fluid dynamics of real tumor tissues. The laminar and continuous blood flow simulations do not fully capture complex, pulsatile and turbulent nature of real blood flow, tumorous branching vessels. The input dataset of the models is solely derived from CFD simulations, which are themselves based on assumption and simplifications. Any inaccuracies in the CFD model will propagate into the machine learning predictions. These models are also based on only two or three variables: spatial coordinates x(m), y(m) and velocity U(m/s). Other influential factors like blood viscosity variations, particle interactions or biological responses are not considered. Studies from literature have been used in some studies to validate the models, but lack of *in vitro* and *in vivo* experimental validation limits the applicability to real world biomedical scenarios. Over-reliance on CFD simulations for a dataset can lead to overfitting and poor generalization to real biological systems. In future, generalization of this models to 3D tumor geometries, flow conditions, or patient-specific data should be considered to increase the credibility of the ML predictions. Multi-comparison of training and testing models and optimization algorithms should be adopted to assess computational efficiency of the models. While runtime is mentioned, there is no discussion on scalability, parallelization, or resource optimization, which are critical for clinical deployment.

Many individual experimental studies fail to produce generalizable evidence regarding NP efficacy and safety, primarily due to biological variability, high costs, and ethical concerns associated with in vivo testing. This restricts the prioritization of informative experiments and slows progress in the field [110]. Despite decades of research, only six nanocarriers (all liposomal) have received FDA approval since 1995, highlighting the low clinical translation efficiency due to an incomplete understanding of nanocarrier/biological interactions [109]. A major bottleneck is the lack of standardized computational frameworks that integrate Computational Fluid Dynamics (CFD) and Machine Learning (ML) for NP design, tumor targeting, and tumor microenvironment reconstruction. The absence of harmonized ML-CFD-NP models delays their adoption in translational cancer research [111]. Future modeling efforts should incorporate tumor evolution and immune responses, enabling temporal analysis of drug-tumor interactions and improving predictive accuracy. Additionally, the heterogeneity of the extracellular matrix (ECM), including fiber orientation and density, plays a critical role in NP transport and retention, and must be considered in model development [112], [113], [114]. The core argument for integration is powerful, asserting that combining these methods allows researchers to leverage the interpretability of physical principles while benefiting from the speed and comprehensiveness of ML models, ultimately addressing complex transport barriers in the body [109].

## 5 Conclusion and future prospects

Drug delivery systems and tumor treatment evaluated using real images is important in personalized treatment. Intravascular and interstitial fluid flow should be investigated together for a more complete and realistic cancer nanomedicine study. Multiscale methods to investigate the effect of the host vasculature and tissue structure on delivery efficacy of nanoparticles and hence identify the optimal physiological conditions for size independent therapeutic drug delivery. There is still a need for more detailed understanding of the molecular mechanisms of nanoparticle transport to and in the tumor. Combined with effective design strategies that takes into consideration patient data and all possible nanoparticle physicochemical attributes have the potential to improve the efficacy of nanoparticle-based drugs in cancer treatment. These studies should include more experimental validation of the simulations to assert results consistency. Drug-delivery is a complex issue which requires an interdisciplinary effort including *in vitro* and *in vivo* experiments and realistic numerical simulations.

CFD simulations and models adopt pre-determined constant values for material properties such vascular permeability and tissue hydraulic conductivity and this does not model the inter- and intra-patient heterogeneity of tumor components. There is need to extend this modeling approaches or simulations in a patient-specific and clinically feasible scenario. This can be achieved through CFD analysis of quantitative MRI data to characterize the tumor-nanodrug interaction on a patient-specific basis. These investigations should account for arbitrary tumor geometries. In the future CFD simulations should also include advanced nanoparticles such as smart responsive NPs and diverse tumor types such as hematological malignancies to broaden this research area. This would make the article more comprehensive. Mapping flow fields within the tumor theorizes a more precise understanding of flow through tumor-associated vessels and the interaction with the surrounding interstitial to provide critical insight into the delivery of therapies. The main goal of cancer therapy is to achieve the highest fraction of killed cells for tumor removal and minimal side effects experienced in healthy tissues. CFD and nanomedicine collaboration provides a system that accounts for multiscale phenomena, which is validated by biomedical engineers and scientists, as well as oncologists working together to develop a modeling framework that advances personalized, effective cancer treatment.

While currently ML models are used to validate experimental data, in light of saving computational expenditure (CFD) and prolonged nanoparticle, it can be adopted as the first point of action, followed by validation with CFD and experimental work. ML will serve as high high-throughput screening of relevant input features for nanoparticle design and tumor parameter before extensive preclinical experiments. This approach can reduce, refine, and partially replace animal experimentation in cancer nanomedicine research. Future models could be improved by developing multi-task models that simultaneously evaluate multiple responses (e.g., pharmacokinetics, DE, and toxicity) to improve generalization and overfitting. If researchers could make it mandatory to incorporate experimental data to validate and refine the models, specify nanoparticle characteristics to improve reproducibility and interpretation, expand models to include dynamic changes in TME, explore combination therapies and multiphase delivery strategies, and link simulations and their predictions to clinical outcomes. This would enhance CFD-ML-NPs hybrid models' advancement into clinical translation to develop effective targeted and personalized cancer treatment.

## 6 Limitations of the study

While this literature review provides a comprehensive overview of existing research on the efforts of CFD models in modeling targeted NP delivery, several limitations should be acknowledged. First, the selection of sources was limited to peer-reviewed articles published in English, which may have excluded relevant studies in other languages or formats, such as dissertations or grey literature. This language and publication bias could potentially narrow the scope of findings. The review focused primarily on studies published within the last 10 and a half years (2014–2025) to ensure relevance and currency. However, this temporal constraint may have overlooked foundational or seminal works that continue to influence the field. While efforts were made to include diverse perspectives, the review may reflect a disproportionate emphasis on certain methodological approaches due to the availability and accessibility of literature. This could affect the generalizability of the conclusions drawn. Finally, the synthesis of findings was based on thematic analysis rather than meta-analysis, which limits the ability to quantify effect sizes or statistically compare outcomes across studies. Future research could benefit from a more systematic or quantitative approach to better assess the strength and consistency of evidence.

## CRediT authorship contribution statement

**K.M Mmereke:** Writing – review & editing, Writing – original draft. **A.O Adewale:** Methodology, Conceptualization. **T. Masebe:** Writing – review & editing. **F. Nemavhola:** Writing – review & editing, Funding. **T Pandelani:** Writing – review & editing, Writing – original draft, Formal analysis, Funding, Conceptualization.

## Author statement

We confirm that the manuscript has been read and approved by all named authors and that there are no other persons who satisfied the criteria for authorship but are not listed. We further confirm that the order of authors listed in the manuscript has been approved by all of us.

We understand that the Corresponding Author is the sole contact for the Editorial process. He is responsible for communicating with the other authors about progress, submissions of revisions and final approval of proofs

## Funding

This work was supported by the National Research Foundation of South Africa, NRF Grant Number: CSUR240511218301.

## Declaration of Competing Interest

The authors declare that they have no known competing financial interests or personal relationships that could have appeared to influence the work reported in this paper.

## Data Availability

No datasets were generated or analyzed during the current study.
