# Supplementary material for: Computational fluid dynamics modeling and simulation of nanoparticle-tumor interaction: Systematic literature review
Source: Comput Struct Biotechnol J. 2025 Nov 12;29:328–52. doi: 10.1016/j.csbj.2025.11.013 (PMC12682063; doi:10.1016/j.csbj.2025.11.013)
Supplement: Supplementary file 1 — Supplementary material [file mmc1.docx]

DESCRIPTIVE STATISTICAL ANALYSIS OF THE (RESULTS AND DISCUSSION)

***Methodology***

References of the study were exported from RefWorks citation manager as a BibTex file for bibliometric analysis. The analysis was performed on R-studio version *2024.12.0.467* by using the bibliometrix package.

***Results***


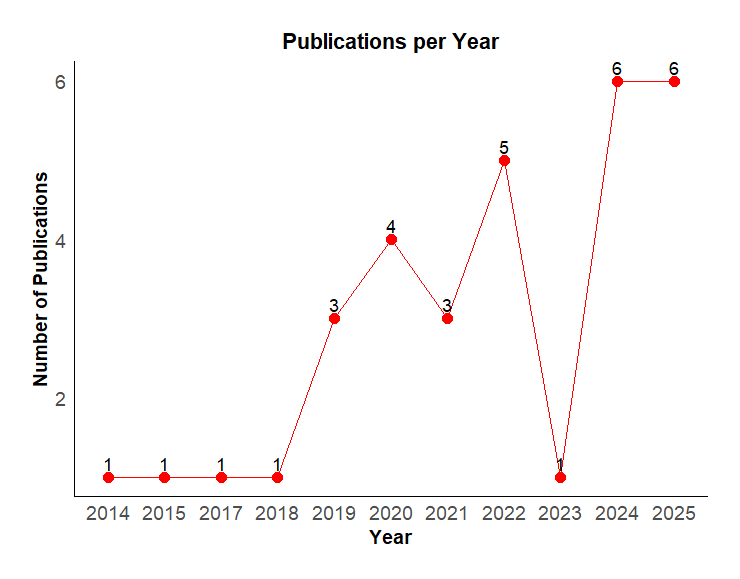


Figure 3: Article production for each year from 2014 to 2025


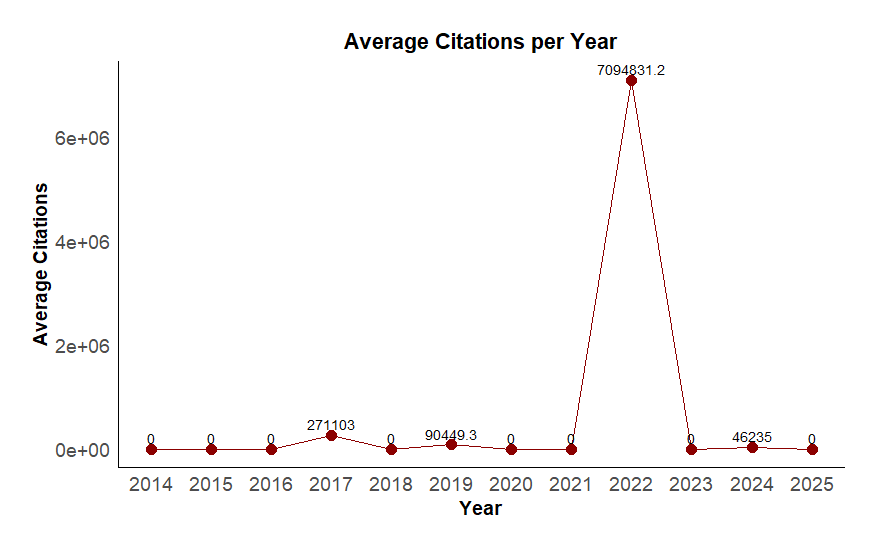


Figure 4: Mean citation of studies grouped by year

The average number o citations per document for each year is shown in figure. Each year has a labelled value showing average citations. From 2014-2016, 2018, 2020-2021 and 2025 the citations are zero. However, 2017 and 2019 shows an increase in average citations; 271 103 and 90 449.3 respectively. In 2022, there is an extreme spike to 7,094,831.2 which is highly unusual compared to other years. After 2024, average drops sharply to 46,235.


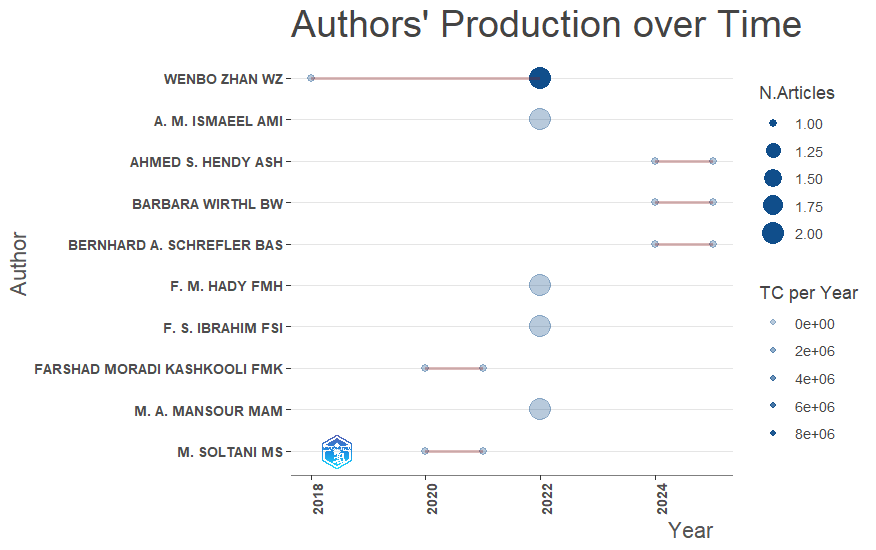


Figure 5: Number of documents each author has published across the years 2014-2025 along with their citation impact.

This graph shows a timeline of publications and the authors over the years. Each dot represents an author’s publication in a specific year and the size of the dot corresponds to the number of articles published that year. The larger dots represent more articles. The horizontal line connects the years in which the author published, showing their activity span. The color intensity indicates Total Citations per year where darker dots imply a higher citation impact.


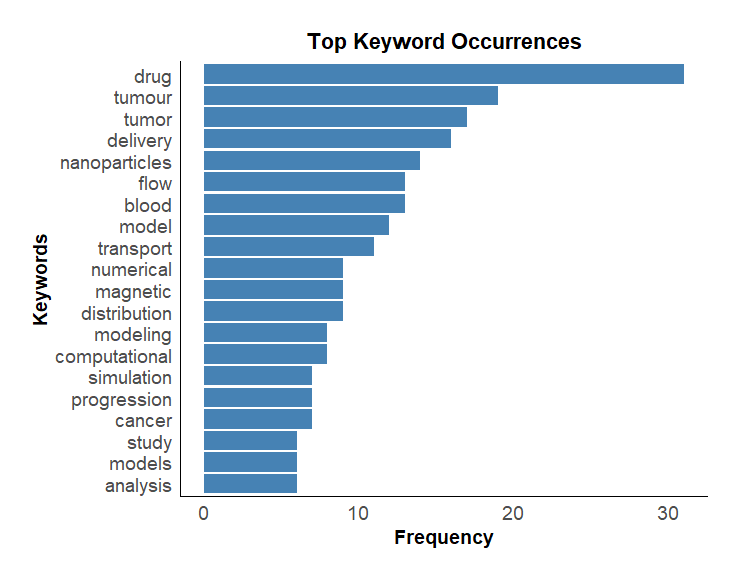


Figure 6: A horizontal bar plot showing the most frequently used keywords in the analysed studies.

“Drug” is the most frequent keyword, appearing about 30 times, suggesting a strong research focus on drug-related topics. “Tumor” and “Tumour” both frequently, approximately 20 times each indicating that the studies are related to cancer, which it true to the aim of the review.


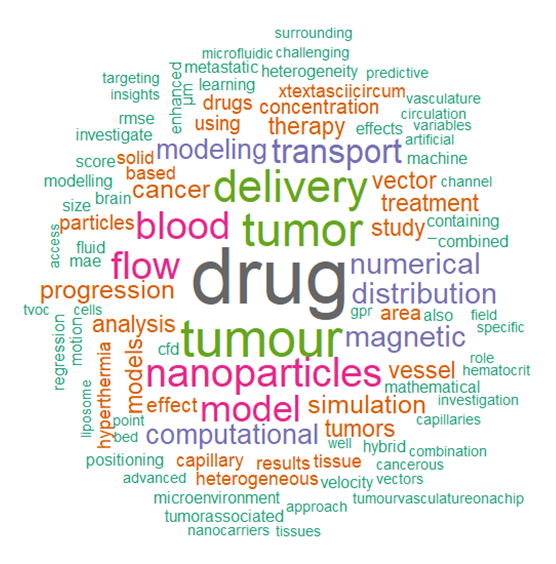


Figure 7: Word-cloud showing key term occurrences in the studies.

Figure shows the word-cloud of keywords in the studies. Largest words (drug, tumor, tumour, delivery, flow, blood, nanoparticles, model, simulation) show most frequent keywords. Medium sized words (therapy, computational, numerical, and magnetic). Smaller words (hyperthermia, heterogenous, capillary, microenvironment) represent a niche or emerging topics.


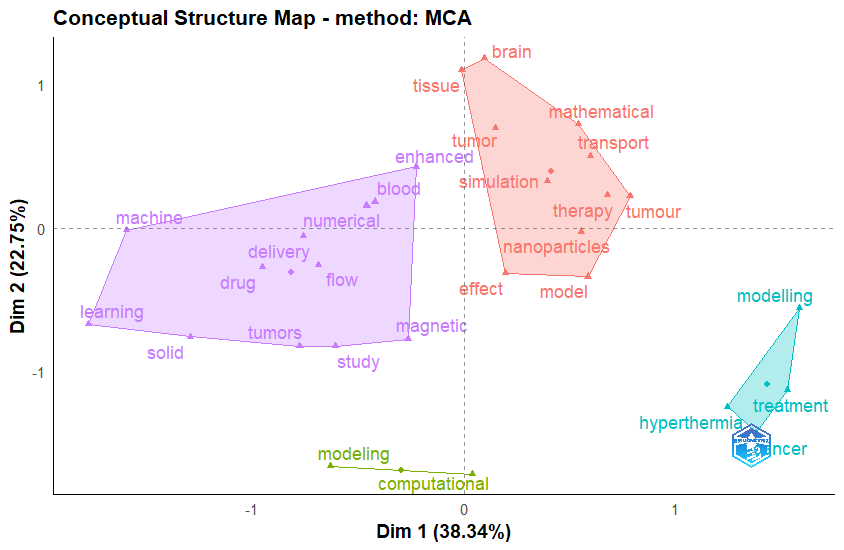


Figure 8: Visualisation of thematic clusters of keywords in the studies

Figure: shows a Conceptual Structure Map created using Multiple Correspondence Analysis (MCA) in bibliometric analysis. The keywords are based on their co-occurrence in the dataset to help identify the major themes and how they relate to each other. The clusters show groups of keywords that frequently appear together, the closer keywords are on the map, the more related they are conceptually. Larger clusters indicate broader themes while smaller clusters indicate niche topics.


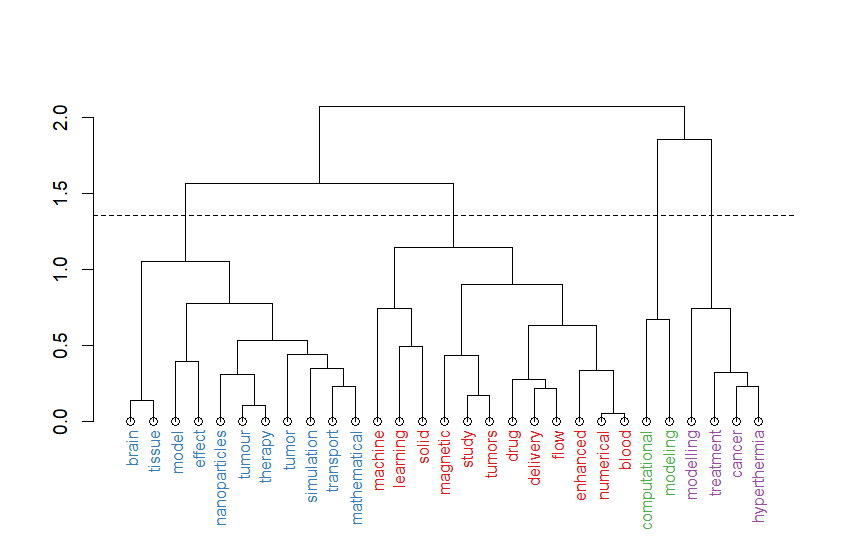


Figure 9: Hierarchical clustering dendrogram of keywords

Figure is a display of keywords, commonly used in bibliometric analysis to show conceptual relationships between terms. The dendrogram groups words based on their similarity or co-occurrence in the dataset. The bottom (leaves) shows individual keywords as described from the dataset. Branches show how keywords cluster together into different thematic groups that are represented by colour. Shorter branches show stronger similarity between keywords while longer branches show weaker similarity.

***Discussion***

A single or few documents published in 2022 might have received massive citations, skewing the average or the inclusion of highly influential papers. The huge spike (>7 million) in 2022 makes smaller values almost invisible on the plot. The dataset includes only partial data for 2025 publications hence the low average citations, so the point is near zero. The line and point for 2025 are present, but visually compressed due to extreme outlier in 2022. It is clear from the authors production over time that Wenbo Zhan has the largest and darkest dot size indicating that they are the most productive and highly cited in 2022.Other authors such as M. Soltani have smaller dots spread across multiple years indicating consistent but lower outputs. Barbara Wirth and Benhard A. Schrefler show multiple years of activity but smaller citation impact.

The studies are related to medical or pharmaceutical research, with emphasis on drug delivery, cancer treatment, and computational modelling. High-frequency keywords include delivery, nanoparticles, flow, and blood point out themes like drug delivery systems and biomedical research. Lower frequency keyword such as analysis, models, and study suggest methodological aspects are also present but less dominant. The presence of both “tumor” and “tumour” shows variations in spelling. This could be standardized for better analysis. Large size words suggest the main research focus is on drug delivery, tumor modelling, and nanoparticle-based therapies. The medium size words suggest secondary themes like computational modelling and magnetic drug targeting. Therefore, the research is heavily focused on drug delivery systems for tumors, simulation and modelling techniques as well as nanoparticle applications. Computational and numerical methods are also significant, suggesting a strong link to bioengineering and computational biology.

The red cluster has keywords that are likely related to tumor modelling and therapy, the purple cluster keywords are related to machine learning and computational fluid modelling; the blue cluster words are more focused on cancer treatment and hyperthermia. Finally, the green cluster is focused on general computational modelling theme. The dendrogram agrees with the conceptual framework as they show similar thematic groups and keywords. The blue cluster is related to tumor biology and therapy, red cluster is linked to machine learning and drug delivery systems while the green cluster is focused on computational modeling and cancer treatment.
